# Design of functional biomaterials as substrates for corneal endothelium tissue engineering

**DOI:** 10.1093/rb/rbac052

**Published:** 2022-07-29

**Authors:** Begona M Bosch, Elia Bosch-Rue, Marina Perpiñan-Blasco, Roman A Perez

**Affiliations:** Bioengineering Institute of Technology (BIT), Universitat Internacional de Catalunya, Barcelona 08195, Spain; Basic Sciences Department, Universitat Internacional de Catalunya, Barcelona 08195, Spain; Bioengineering Institute of Technology (BIT), Universitat Internacional de Catalunya, Barcelona 08195, Spain; Basic Sciences Department, Universitat Internacional de Catalunya, Barcelona 08195, Spain; Bioengineering Institute of Technology (BIT), Universitat Internacional de Catalunya, Barcelona 08195, Spain; Basic Sciences Department, Universitat Internacional de Catalunya, Barcelona 08195, Spain; Bioengineering Institute of Technology (BIT), Universitat Internacional de Catalunya, Barcelona 08195, Spain; Basic Sciences Department, Universitat Internacional de Catalunya, Barcelona 08195, Spain

**Keywords:** corneal endothelium, tissue engineering, cell therapy, biomaterials

## Abstract

Corneal endothelium defects are one of the leading causes of blindness worldwide. The actual treatment is transplantation, which requires the use of human cadaveric donors, but it faces several problems, such as global shortage of donors. Therefore, new alternatives are being developed and, among them, cell therapy has gained interest in the last years due to its promising results in tissue regeneration. Nevertheless, the direct administration of cells may sometimes have limited success due to the immune response, hence requiring the combination with extracellular mimicking materials. In this review, we present different methods to obtain corneal endothelial cells from diverse cell sources such as pluripotent or multipotent stem cells. Moreover, we discuss different substrates in order to allow a correct implantation as a cell sheet and to promote an enhanced cell behavior. For this reason, natural or synthetic matrixes that mimic the native environment have been developed. These matrixes have been optimized in terms of their physicochemical properties, such as stiffness, topography, composition and transparency. To further enhance the matrixes properties, these can be tuned by incorporating certain molecules that can be delivered in a sustained manner in order to enhance biological behavior. Finally, we elucidate future directions for corneal endothelial regeneration, such as 3D printing, in order to obtain patient-specific substrates.

## Graphical Abstract

**Figure:**
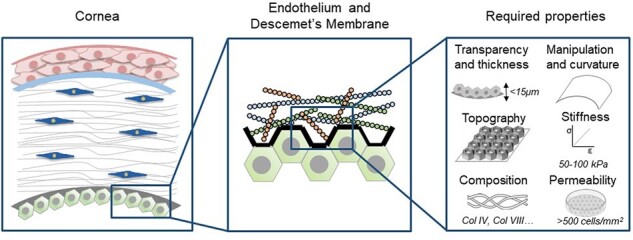

## Introduction

Diseases affecting the cornea are the fifth leading cause of blindness affecting more than 10 million people in the world [1, 2]. Nowadays, corneal transplant is the gold standard treatment for these diseases. In fact, it is the most frequent type of transplant performed worldwide [3].

**Figure 1.**
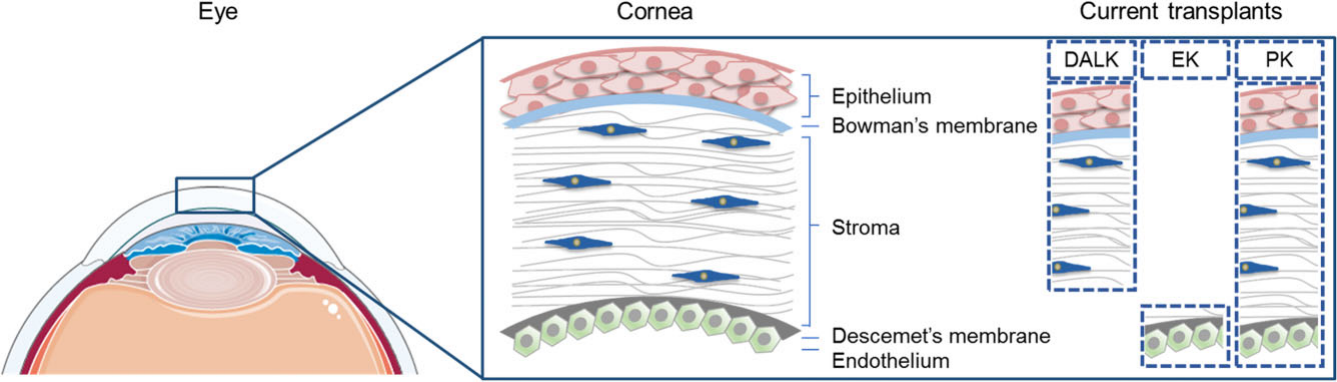
Anatomy of the eye and cornea. The cornea is the outer layer of the eye and contains different layers: epithelium, Bowman’s membrane, stroma, Descemet’s membrane and endothelium. Current transplant are DALK replaces posterior layers; EK replaces anterior layers; and PK replaces the entire cornea. DALK, deep anterior lamellar keratoplasty; EK, endothelial keratoplasty; PK, penetrating keratoplasty.

An organ transplant is the replacement of a dysfunctional tissue or organ with a healthy one that can be from the same patient (autograft), from a human donor (allograft) or an animal (xenograft). Corneal transplant, named as keratoplasty, can be performed using two different techniques, mainly replacing the full cornea, known as penetrating keratoplasty (PK), or only replacing the damaged corneal layers, known as partial thickness transplants [4]. PK replaces the five layers of the cornea, whereas partial thickness transplants conserve the healthy and functional tissue from the cornea, presenting mainly two types of transplants, being the deep anterior lamellar keratoplasty (DALK) and the endothelial keratoplasty (EK). DALK replaces the anterior layers (epithelium, Bowman’s membrane and stroma), whereas EK selectively replaces the posterior layers (endothelium, Descemet’s membrane and, in some cases, part of the stroma; Fig. 1). Over the last decade, the number of PK has been decreasing and partial thickness transplants are the most selected option. In fact, according to the annual Eye Bank Association of America (EBAA) report from 2021, EK was the most performed keratoplasty, representing more than 60% of all corneal transplants [5]. The top indication for corneal transplant is related to Fuchs dystrophy, a disease that affects the corneal endothelium, which represents 39% of all transplants performed [3].

Despite of the great advances in the clinical scenario, corneal transplantation still faces several unmet challenges, such as donor shortage and rejection, that limit the overall viability and urges the seek of alternative solutions [3, 6]. Taking into account that the most damaged and vulnerable tissue is the endothelium, there is a need to find alternative solutions such as the development of an *in vitro* tissue construct for corneal endothelium regeneration.

The main difference of the corneal endothelium with other tissues or cell layers in the human organs is that corneal endothelial cells (CEC), upon external aggression or aging, do not present regenerative capacity. This is mainly related to their non-proliferative state, leading to a decrease in functionality, reducing the transparency and allowing the formation of edema. Subsequently, this edema can result in a pathological condition named as bullous keratopathy, requiring a surgical treatment in order to recover the corneal dysfunction [7].

Current alternatives are based on stem cell therapies that can be combined with synthetic matrixes. Cell therapies have gained interest as there are several routes that may allow the production of CEC. Once these CEC are produced, these need to either assemble among them forming a cell sheet that can be directly implanted, or to be combined with extracellular matrices (ECM) materials for its subsequent implantation. In the latter case, these cells need to be in direct contact with a substrate similar to the Descemet’s membrane (DM) and attach onto it. Hence, in order to replicate the native scenario, biomatrixes are being designed with several assets similar to the DM that will allow a better interaction and will, at the same time, allow direct implantation of a construct composed of biomaterials and cells.

Here, we aim at describing the current alternatives to corneal endothelial transplantation, taking special consideration in the design of biomaterials that can provide a natural support for CEC. In this sense, biomaterials can have two pivotal roles, allowing on the one hand, the production of cell sheets to allow direct implantation in a scaffold-free system or the combination of cells with adequate biomaterials. In both cases, biomaterial design will play a key role in providing the stimuli required for cells to produce the desired morphologies and functionalities.

## Required properties for corneal endothelium regeneration

**Figure 2.**
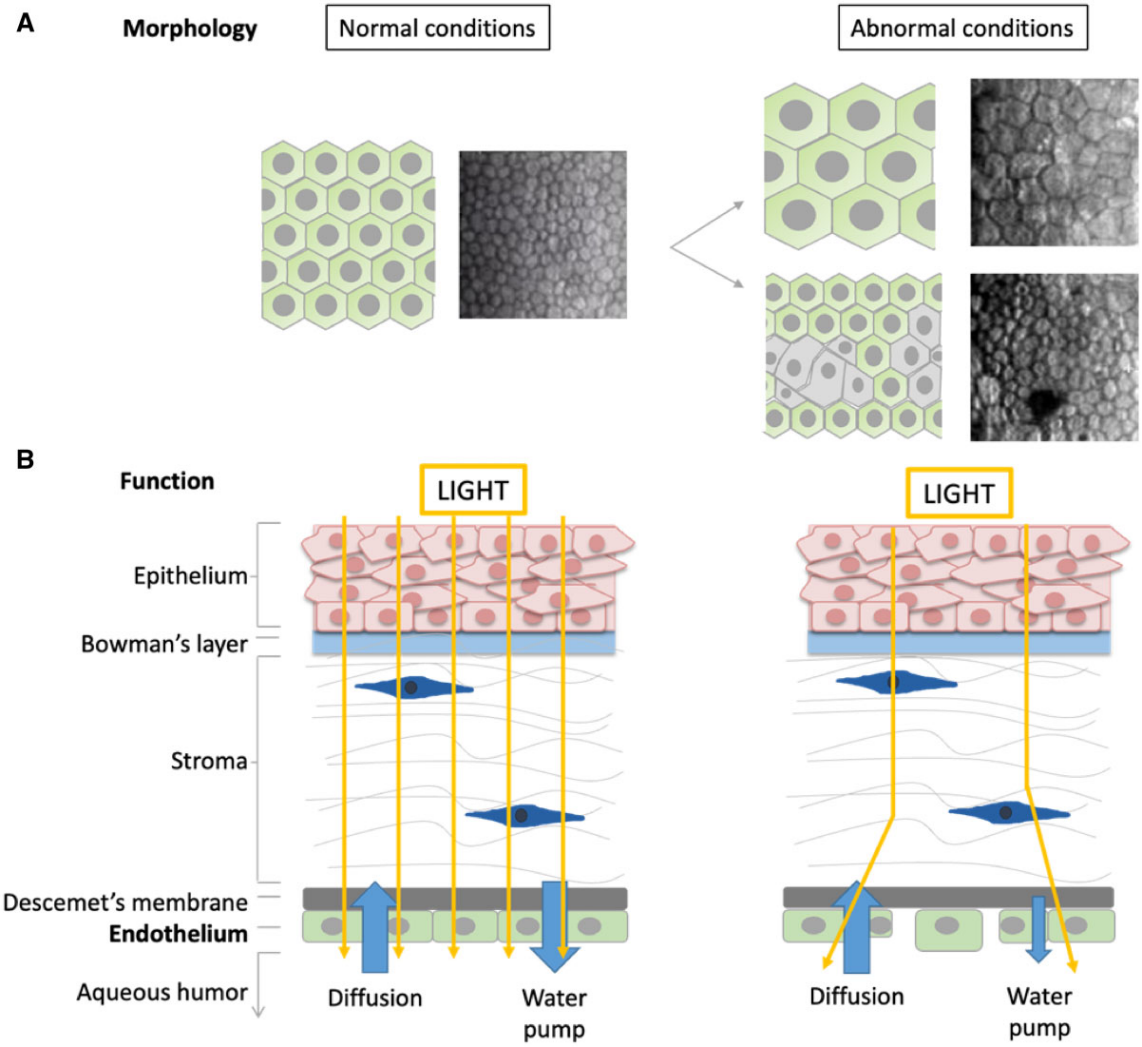
Corneal morphology and functionality in normal and abnormal conditions. Cells present an hexagonal shape in normal conditions whereas its shape and size its modified in abnormal conditions (**A**). Light can enter through and arrive to the inner parts of the eye in normal conditions but light is not able to arrive in abnormal conditions (**B**). Adapted with permission from Ref. [8].

Native tissues present several conditions that allow the different functionalities to work properly. The different functionalities refer to the main characteristics and task each tissue should perform. For instance, bone is a tissue that provides structural support to the body, hence, having a damaged bone, may limit the structural support of the body. In the case of the cornea, and more specifically, the corneal endothelium is mainly related with a proper visualization, hence having alterations in the corneal endothelium may limit the visibility as well as induce visual impairment or even blindness. Nevertheless, whenever there is damage and the cells are found in abnormal conditions, normal functionalities are lost (Fig. 2). Abnormal conditions generally take place either due to an increase in cell size or to a change in cell morphology that may ultimately reduce the properties required for the proper functionality (Fig. 2).

**Figure 3.**
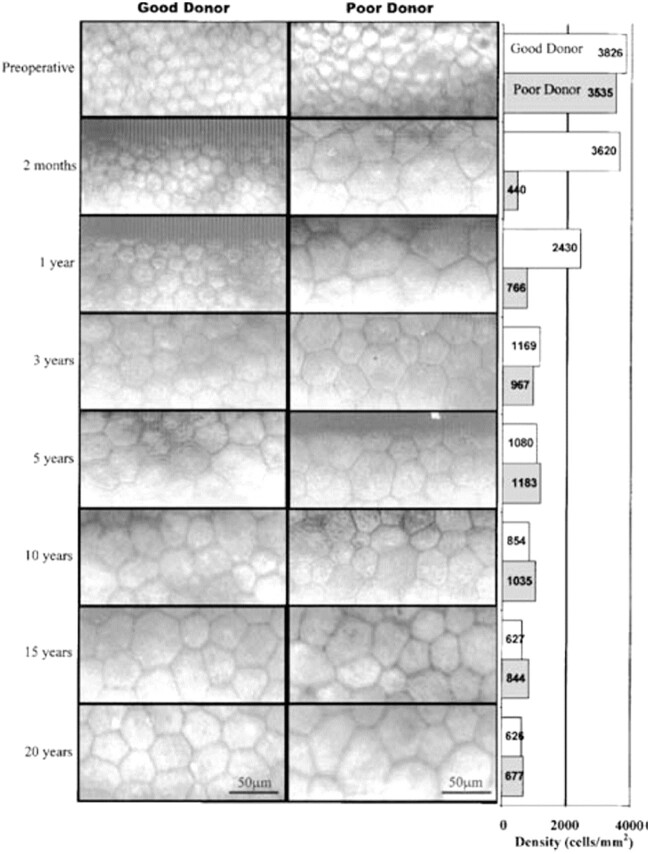
Morphology of the corneal endothelium depending on the quality of the cells and the age of the donor. Adapted with permission from Ref. [9].

CEC are of great relevance and, hence, these need to be carefully examined to evaluate the need of transplantation. Eventually, these CEC, in the case of deceasing, can be a potential cell source to be transplanted to a patient requiring corneal endothelium replacement. For this purpose, CEC quality has been deeply studied, showing that age significantly affects cell quality and proper donors need to be carefully selected. In this sense, abnormal cell morphology (Fig. 3) may lead to non-functional corneal endothelium which limits vision capacity [9].

**Figure 4.**
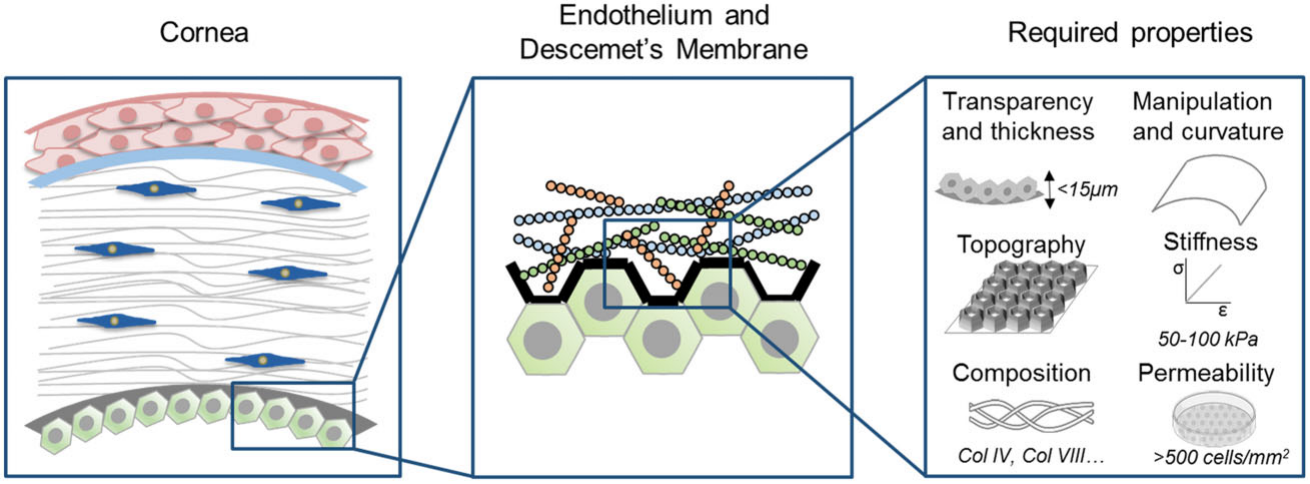
The anatomy of the cornea. The cornea is the outer layer of the eye and contains different layers. The inner layer is the corneal endothelium, which is in direct contact with the Descemet’s membrane and needs specific properties to have a correct functionality.

In order to maintain the properties as similar as possible to native tissue, we initially provide an overview of the different aspects related to the corneal endothelium in order to take this into account when designing materials for its replacement. There are several parameters that define normal behavior of the corneal endothelium, which are listed below (Fig. 4).

*Transparency* refers to the ability of light to go through the corneal endothelium. CEC play an essential role in the maintenance of corneal transparency through its barrier and the Na^+^/K^+^-ATPase pump functions that regulate corneal hydration [10]. The normal function of corneal endothelium is achieved with an optimum cell density, considered as 2500–3000 cells/mm^2^ [11]. An abnormal pump function results in an excess of fluid in the corneal stroma due to fluid absorption from the aqueous humor. This excess of fluid in the cornea changes the composition of the proteins, which ends in corneal edema and, subsequently, in a decreased level of corneal transparency [10].

*Permeability* is the ability to allow the flow of nutritional elements through the CEC. As cornea is an avascular tissue, the corneal endothelium represents a nutritional gateway between the anterior chamber and the cornea. Most of the nutrients required for the cornea, which are allocated in the aqueous humor, are transferred into the cornea through CEC pumps [12]. In order to maintain this nutritional function, a minimum cell density of 400–500 cells/mm^2^ is required [13].

*Mechanical stiffness* refers to the stability, flexibility and rigidity of the layer that will allow to properly maintain the shape of the layer. The different layers of the cornea have shown values of elastic modulus between 50 and 100 kPa in humans [14]. Nevertheless, the corneal endothelium has shown significantly lower levels, ranging between 5 and 10 kPa in rabbits [15]. Hence, the mechanical stability of the corneal endothelium is not strictly relevant, as the structural mechanical properties seem to be based on the other layers of the cornea. The main component of the DM is collagen, so using collagen as a substrate while providing a stiffness that is comparable to the native environment of CEC seems as a promising approach. In fact, Gutermuth *et al*. [16] evaluated that seeding cells onto smooth collagen enhanced its functionality compared to a silicone substrate.

*Topography* refers to the presence of surfaces with determined roughness that are able to provide physical cues to the attached cells. Topography is among the leading physical cues to guide stem cell fate [17]. Different morphologies have been proposed, which can be prepared by replica molding or by stereolithography [18]. A recent study analyzed the microtopography of a DM using 3D confocal microscopy [16]. This consisted of flat hexagonal pits with an irregular morphology, which ends in the formation of the characteristic polygonal or hexagonal morphology of CEC [11]. Their width ranged from 10 to 20 µm and their maximal height was 1 µm [16]. The results showed that mimicking the native structure of DM allowed enhanced CEC functionality. Interestingly, the topography of the DM plays a key role as well in determining the fate of Fuchs’ endothelial dystrophy, which is based on the presence of microtopography on the DM [19]. Previous findings showed that when culturing cells on different topographical features, CEC tended to form monolayers on lower height and density substrates, whereas there seemed to be a reduction in the capacity to form CEC monolayer in the densely packed substrates. Hence, topographical features can as well be used to predict the ability of CEC to form the needed continuous monolayer [19].

*Composition* refers to the vast combination of proteins and polysaccharides present in the cornea that are able to provide the chemical cues and mechanical stability of the matrix. In general, basement membranes are rich in type IV collagen (COL IV), but more specifically, the DM also contains type VIII collagen (COL VIII) that perform a hexagonal pattern [20, 21]. Furthermore, the DM also contains laminin, nidogen, entactin and some glycoproteins and proteoglycans [22].

*Thickness* (although related with stiffness and transparency): DM thickness increases through age, its values vary from 3 µm at birth and arriving to 10 µm in adults [23]. Normally, when performing DMEK, the graft is usually between 10 and 15 µm.

*Manipulation in surgery:* Graft orientation is very important, as CEC have to be in contact with the humor aqueous to be functional. The ‘up-side-down’ graft placement should be avoided [22].

*Curvature:* The internal part of the cornea has a circular diameter of 11.7 mm and a sagittal height of 2.18 mm to the center of the cornea [23]. Interestingly, a corneal map has been depicted in order to understand how the curvature affects the healing of the cornea. This may provide relevant information on how to design biomaterials with specific curvatures in order to adjust the tension produced by the curvature [24, 25]. For instance, an *in vitro* model using a radius of 8.4 mm and an angle of 100° allowed stimulating cell alignment and consequently enhance the human corneal tissue equivalents [26].

## Sources of therapeutic cells

In order to provide a functional corneal endothelium, the presence of cells is necessary. As previously described, these cells have limited availability from donors and for this reason, it is of great importance to find alternative sources, which should be from the autologous origin where possible, that may allow replicating similar cell structures. In this sense, corneal endothelial regeneration using cell therapies is based on the isolation and expansion of human CEC (HCEC) or the differentiation of stem cells into CEC.

### CEC culture conditions

It has been reported that the modulation of phosphatidylinositol 3-kinase (PI3K)/Akt and Smad2 signaling pathways promotes CEC proliferation and wound healing [27]. Hence, previous research has evaluated the effect of different molecules in cell culture medium as potential modulators of the mentioned pathways.

On the one hand, epidermal growth factor (EGF), basic fibroblasts growth factor (bFGF) and transforming growth factor beta 2 (TGF-β2), which are present in the endothelium and the aqueous humor, have been described to enhance *in vitro* cell migration, proliferation and corneal wound healing [27]. This can be explained as EGF and bFGF activate PI3K/Akt pathway, which permits CEC to enter in the proliferative S-phase of the cell cycle [28–32]. Differently, TGF-β2 induces the suppression of the cell proliferation. Despite of this suppression of the proliferative status, TGF-β2 is able to enhance cell migration and corneal endothelial wound healing through the Smad2 pathway.

On the other hand, it has been reported that insulin growth factor 1 (IGF-1), heregulin beta and activin A permits an upregulation of PI3K/Akt and Smad2 signaling pathways, which promote CEC proliferation, migration and physiological functions. In this sense, these factors were shown to successfully recover the corneal thickness in a rabbit model [33, 34].

Moreover, it has been demonstrated that the Wnt/β-catenin pathway is also related to CEC proliferation. Specifically, through the expression of WNT10B, which activates the Cyclin D1 that is implicated in HCEC proliferation [35]. Therefore, the incorporation of molecules that activate this pathway, such as IL-1β or a GSK3β inhibitor, are also used to culture HCEC [35].

Although culture components are indispensable for the correct formation and expansion of CEC, the culture methodology is also a very important factor to consider. Cells can be cultured in adherent or in suspension conditions. Most cell types are cultured as a cell monolayer in adherent culture, using treated-culture plates to enhance its adhesion and proliferation. Cells can also be cultured in suspension conditions, which forms a 3D cell culture system that mimics the physiological tissue environment and permits a higher cell-to-cell interaction. It has been demonstrated that the formation of 3D aggregates in suspension culture of stem cells resembles an early-embryogenic process, known as gastrulation, which permits the formation of a more primitive stage of differentiation and, subsequently, higher differentiation potential [36].

The main cell source for the formation of large number of HCEC is primary cells or differentiated stem cells. Primary cells are isolated from human cadaveric donors and expanded *in vitro*. These cultured cells are able to proliferate with signaling molecules, which can then be expanded to reach a sufficient cell number, allowing direct CEC transplantation [37–39]. However, the limited availability of this cell source limits its clinical use. For this reason, human stem cells from diverse origins have been evaluated, such as pluripotent stem cells (PSC), corneal stroma, skin, umbilical cord, bone marrow, adipose tissue and dental pulp. In the following sections, we will briefly describe how these cells from other sources can be used to obtain CEC.

### CEC differentiation

#### Pluripotent stem cells

Different protocols have been used for the formation of CEC from PSC such as embryonic stem cells (ESC) or induced Pluripotent Stem Cells (iPSC). For instance, Chen *et al*. [40] generated CEC-like cells from ESC using two different conditioned culture medium. This process was regulated by the TFG-β2 signaling pathway. Their results showed that CEC-like cells presented the typical hexagonal morphology and were able to express CEC-specific markers such as N-cadherin, Na^+^/K^+^-ATPase, ZO-1 and vimentin at protein and RNA level [40]. Other study generated CEC-like cells in two phases [41]. First, ESC was cocultured with corneal stromal cells in a culture medium with EGF and bFGF and generated periocular mesenchymal precursors (POMPs). Second, POMPs were differentiated into CEC-like cells using a conditioned medium. Their results showed that cells expressed CEC markers N-cadherin, FoxC1 and Pitx2, which are crucial in ocular development. Furthermore, CEC-like cells were transplanted and could restore corneal transparency in rabbit corneal endothelium dysfunction models [41]. Moreover, a two-step protocol has also been reported to derive PSC into CEC [42, 43]. For this purpose, Smad inhibitors or small molecules such as a GSK3β inhibitor were used in an initial step, which induced cells to differentiate into neural crest, which is a precursor state similar to the natural formation of CEC. Then, cells were cultured in presence of FGF-2, among others, and were able to generate CEC-like cells. Differentiated cells were similar to native CEC, with a hexagonal morphology and expressing typical markers as ZO-1, Na^+^/K^+^-ATPase, COL8A1 and COL8A2, among others [42, 43]. A recent study was able to generate corneal endothelial cell substitute cells from iPSC (named as CECSi cells) using a direct differentiation protocol [44]. Their differentiation medium contained IGF-1, among others, and iPSC were able to differentiate into CECsi cells in 11–14 days. These CECsi cells exhibited a hexagonal morphology and an increased expression of CEC markers such as Na^+^/K^+^-ATPase, ATP1A1, ZO-1, N-cadherin and Pitx2. Moreover, differentiated cells with ROCK inhibitor were transplanted into a monkey corneal edema model and a recovery of the edema was showed [44].

#### Bone marrow-derived endothelial progenitor cells

Human limb fetal bone marrow has also been used as a source for the generation of CEC-like cells [45]. For this purpose, bone marrow-derived endothelial progenitor cells were transdifferentiated into CEC using a coculture system with fetal CEC with CEC conditioned medium. Transdifferentiated cells expressed aquaporin 1 (AQP1), a CEC marker, and formed polygonal cells. Transplanted cells of a cat model with a corneal endothelium defect recovered corneal transparency after 28 days [45].

#### Corneal stroma precursors

Another study evaluated the use of stem cells from the corneal stroma, known as Corneal-precursors, to differentiate into CEC. Previous work demonstrated that cell differentiation was achieved based on the upregulation of Pitx2, which occurred in the presence of retinoic acid and a GSK3β inhibitor [46]. Differentiated cells were transplanted into rabbit corneas, allowing the maintenance of its transparency and thickness for 8 days [46].

#### Adipose-derived stem cells

A protocol has also been described for obtaining CEC-like cells from autologous adipose-derived stem cells (ADSC). This was performed using an initial step of cell reprogramming and the coculture of ADSC with rabbit CEC. Their results showed that CEC-like cells were positive for CEC markers CD31, AQP-1 and ZO-1 [47].

#### Skin-derived precursors

The dermis contains a population of autologous stem cells that can be easily isolated through a minor surgery. Inagaki *et al*. [48] demonstrated that skin-derived precursors (SKP) from facial skin had the ability to differentiate into CEC-like cells. The differentiation was performed by the activation of Smad2 and Wnt/β-catenin pathway by supplementing the culture medium with, among others, a GSK3β inhibitor and with TGF-β2. Differentiated cells presented a polygonal morphology and were positive for specific CEC markers ZO-1 and Na^+^/K^+^-ATPase. Moreover, pump function in CEC-like cells was higher compared to SKP and 3T3 cells. *In vivo* analysis in a rabbit model of bullous keratopathy revealed that differentiated cells could maintain corneal transparency and thickness [48].

#### Umbilical cord stem cells

Umbilical cord stem cells (UCSC) represent a new allogenic alternative for regenerative medicine and tissue engineering. UCSC cultured with a GSK3β inhibitor showed the ability to differentiate into CEC-like cells. These cells presented typical characteristics like polygonal shape and the expression of CEC markers (ZO-1, Na^+^/K^+^-ATPase and Pitx2). Furthermore, CEC-like cells transplanted into a rabbit model of bullous keratopathy were able to restore corneal transparency and thickness [49].

#### Dental pulp stem cells

Dental pulp tissue is gaining interest due to the regenerative ability of the dental pulp stem cells (DPSC). DPSC can be easily isolated from third molars and represent an autologous cell source. A two-step protocol showed the ability to differentiate DPSC into CEC-like cells. This process was performed through the activation of the PI3K/Akt pathway and the differentiated cells presented the characteristic polygonal-like shape together with an increased expression of typical markers (ZO-1, Na^+^/K^+^-ATPase, COL4A2 and COL8A2) [50].

**Figure 5.**
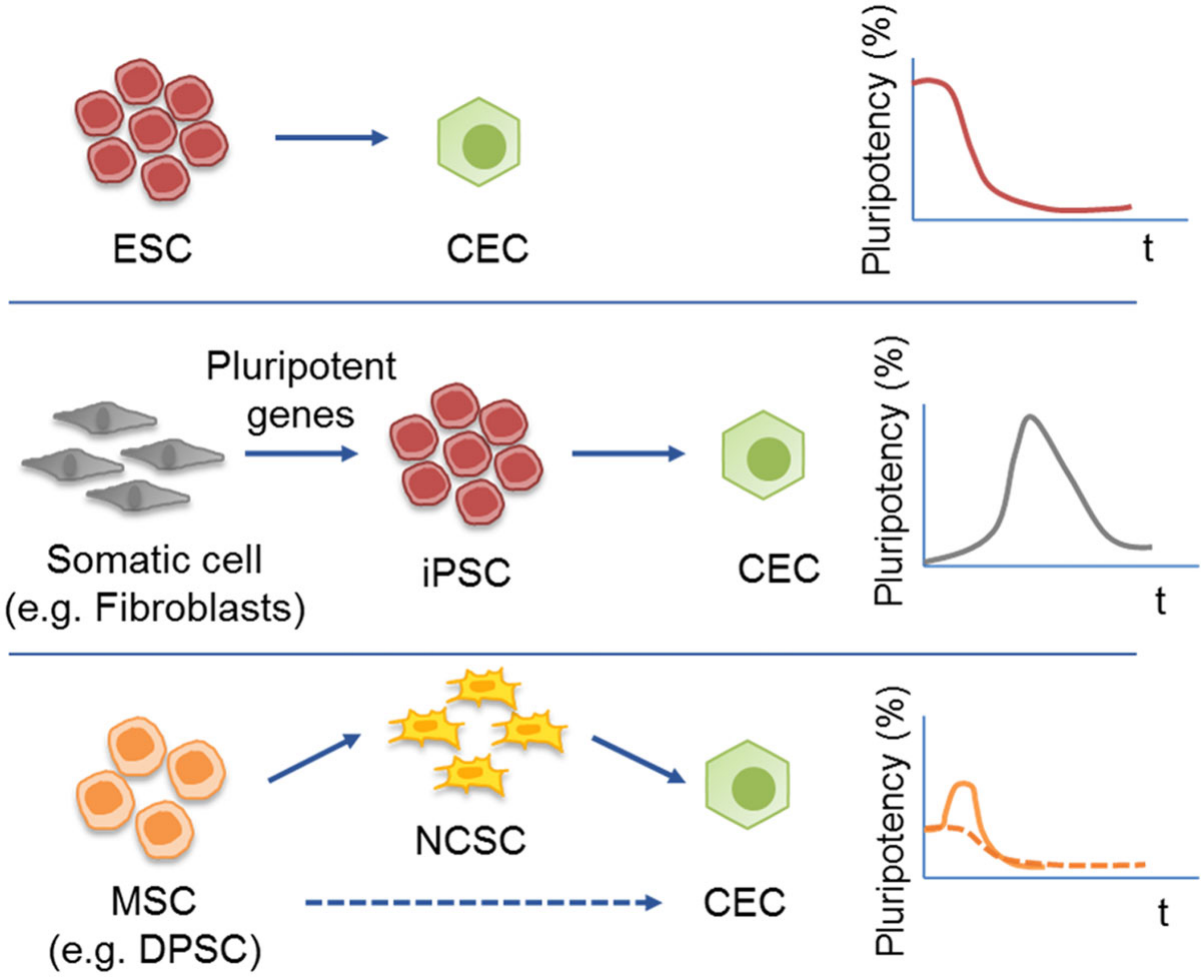
Schematic representation of CEC production depending on the initial pluripotent status of cell source. On top, ESC differentiated into CEC; in the middle, somatic cells (e.g. fibroblasts), can be reprogrammed into PSC, which can be differentiated directly into CEC; at the bottom, MSC can be differentiated directly into CEC or using a two-step protocol. CEC, corneal endothelial cells; DPSC, dental pulp stem cells; ESC, embryonic stem cells; iPSC, induced pluripotent stem cells; MSC, mesenchymal stem cells; NCSC, neural crest stem cells.

To sum up, in general, CEC can be obtained from different sources, depending on their potency and their differentiation level. As shown in Fig. 5, CEC can be differentiated from PSCs (either ESC or reprogrammed iPSC) or from multipotent stem cells.

In general, sources of autologous cells are considered the best choice in order to avoid adverse reactions. Autologous cell sources, such as cells from the dental pulp of the same patients are considered very relevant since this will avoid the possible adverse reaction in the human body as well as the possible immune response. In some cases, cells from other patients or directly from other species are the only possible sources, which entails certain advantages since non-invasive treatments are required in the patients, but may increase the chances of immune rejection and possible complications.

## Materials to promote *in vitro* CEC tissue engineering

### Materials

While the cells previously described are mandatory for the proper reconstruction of the corneal endothelium, biomaterials may provide an adequate environment for cells to perform their function. In general, the number of combinations and types of materials is high, which provides an elevated number of biomaterials that can be used for tissue regeneration and substitution. Depending on the targeted tissue, the type of materials as well as the properties need to be tuned. Not only there are different types of biomaterials available, but a combination of different biomaterials is as well possible, providing an unlimited number of possibilities for tissue engineering. Furthermore, their properties can be further enhanced by incorporating different nano- and microsize morphological features in the materials as well as relevant chemical cues, such as growth factors or molecules that can be released at the desired time point.

Due to the inherent properties previously described for the corneal endothelium, the main types of materials that can be used are described below.

#### Polymers

These materials have been extensively used in tissue engineering due to their excellent properties. They can be chemically functionalized and present unique characteristics as biodegradation, biocompatibility and intrinsic biological domains. They can be classified depending on their origin, a synthetic or natural source [51].

##### Synthetic polymers

These polymers are made of diverse monomers of different lengths. There is almost an unlimited number of synthetic polymers that can be designed depending on the needs. For this reason, they offer the required mechanical and physicochemical properties as tensile strength, stiffness and biodegradation. Normally, synthetic polymers are cheap as they can be easily fabricated in large quantities. Some forms of poly(lactic acid) (PLA) and poly(lactic-co-glycolic acid) (PLGA) have been used in approved products for specific applications by the US Food and Drug Administration (FDA), and therefore could be promising materials to be used in the future as substrates for corneal tissue engineering [52–54].

##### Natural polymers

These materials are present in organism membranes and in ECM and include proteins and polysaccharides. Due to their natural origin, they offer great advantages like biocompatibility and binding domains. These domains help in cell attachment and differentiation as they recognize the natural substrate [55]. For corneal regeneration, collagen is the most important protein as it is the principal component of the cornea. All in all, diverse natural scaffolds have been used in this field [55].

Comparing synthetic and natural polymers, there are two main aspects that distinguish both of them. On the one hand, from a material point of view, the composition and properties of the synthetic polymers can be controlled to a much higher extent compared to the natural polymers. Natural polymers may suffer from batch-to-batch variations, which may alter the final properties of the product. On the other hand, regarding the biological response, synthetic polymers are generally more inert, although strategies can be used to modify or functionalize the surface, whereas natural polymers have the needed amino acids and proteins required for proper cell attachment and cell guidance, being the possible adverse effect the immune response.

#### Composites

The union of a minimum of two distinct materials forms composites. This combination results in an enhancement of physical or biological properties. For example, collagen and chitosan together have been determined to enhance the optical transparency and mechanical strength in membranes for corneal tissue engineering [56].

### Overall morphology

Besides the biomaterial composition, different morphologies of the final biomaterial determine the interaction with the surrounding tissue and the function to be achieved.

### Scaffolds (porous and fibrous)

Three-dimensional scaffolds are the most commonly used structures for tissue engineering. There are several requirements for an ideal scaffold for tissue engineering, which are mainly to provide similar mechanical properties to those of the native tissue, to degrade while the new tissue is being formed and to present sufficient porosity allowing cell infiltration. These scaffolds can be fabricated by several methods, including freeze-drying, solvent casting, solid freeform fabrication or phase separation, among others [51]. A special type of preparation is electrospinning, which allows forming thin layers of nanofibrous materials, mimicking the fibrillar ECM structure [57]. The latter method is of special interest in cornea regeneration as the volume required for CEC culture is relatively low, as only a thin membrane is needed.

### Hydrogels

Hydrogels are polymeric materials that are able to entrap high amounts of water within the polymeric network. The main advantage of hydrogels is that they possess injectability, which is the ability of the materials to be injected into the site of defect [58]. Based on this latter property, the risk of infection in surgery is reduced due to the limited invasiveness of the technique. Furthermore, these materials are prepared at low temperature, which are then able to gelify after injection upon contact with body temperature. This ability further allows hydrogels to encapsulate and deliver biologically active molecules as well as cells [58, 59]. The most commonly used hydrogels are based on polysaccharides and other natural proteins. Their degradation rates are generally fast but can be strategically modified to tune their degradation rates to match with the formation of new tissue.

### Microparticles

Microparticles (MP) are spherical-like particles in the range of tens of microns up to several hundreds of microns that combined with an adequate hydrogel, can be injected in the desired site. These MP are generally used either as microscaffolds onto which cells are able to grow to create a tissue or as systems to allocate molecules and allow the delivery of biologically relevant molecules [60].

### Nanoparticles

In a similar way to MP, nanoparticles (NP) present unique properties based on their nanosizes. Their range of sizes down to the nano level confers them high-specific surface areas with electrostatic charge that allows their penetration into cells [61]. These are generally synthesized by chemical routes and their size ranges between 20 and 100 nm. In the case of cornea, these particles are generally made of polymeric and ceramic materials.

## Biomaterials for producing scaffoldless CEC sheets

Substituting the corneal endothelium consists merely on substituting the thin layer of CEC found in the cornea. With this in mind, one plausible strategy has been the culture of cells in such environment that will enhance the cell-to-cell contact and force the formation of a single-cell layer. This single-cell layer can have the required properties in terms of handling and manipulation for surgeon to implant, as well as the biological requirements needed, as the cells will be maintained alive and hence allowing to perform the previously mentioned functions. For these cell layers to be adequately formed and to allow the proper phenotypic expression of the different markers as well as to easily manipulate the obtained cell sheet, biomaterials have appeared as candidate to enhance the cell-to-cell contact and contribute to the cell sheet formation. In this sense, most of the biomaterials used for this purpose are based on thermosensitive materials that, upon change of temperature, have a transition in the surface properties, transitioning from a hydrophilic material to a rather hydrophobic material that induces cell detachment [62]. Other methods to allow the detachment of cells are based on chemical methods, such as trypsinizing treatment, but their use may limit the maintenance of the cell sheet as well as may limit the phenotypic markers required.

**Figure 6.**
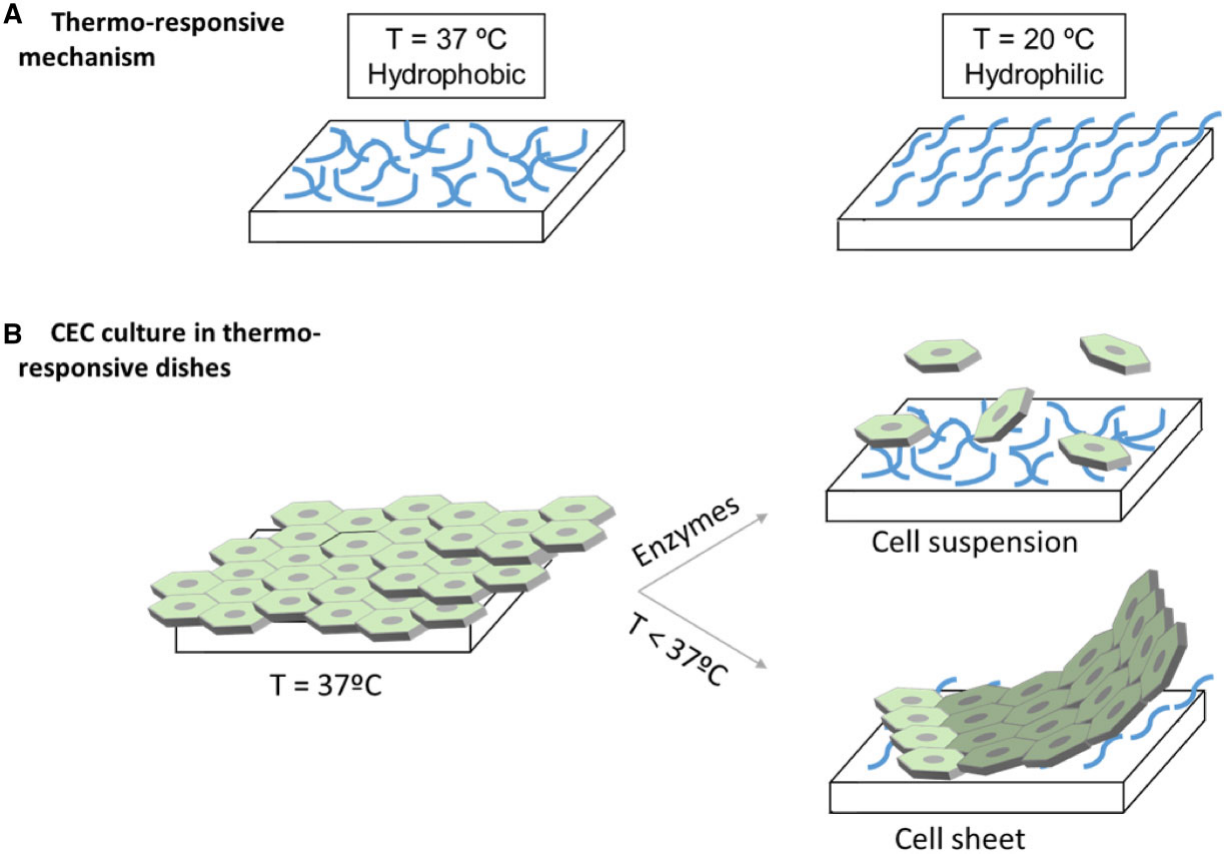
Thermo-responsive dishes mechanism and culture. Thermo-responsive polymers at 37°C act as hydrophobic substrate, whereas at 20°C they act as hydrophilic materials (**A**). Culturing corneal endothelial cells (CEC) in thermo-responsive dishes permits the formation of a monolayer of cells attached to the dish at 37°C that can be isolated through the addition of enzymes as a cell suspension or lowering the temperature and creating a cell sheet (**B**).

One of the most commonly used biomaterials for cell sheet production is the material that can be thermally stimulated for the detachment of adherent cells. In this sense, poly(N-isopropylacrylamide) (PNiPAAm) has been considered as an excellent material for this purpose, as it allows the transition from hydrophilic to hydrophobic based on the temperature (Fig. 6) [62, 63]. Taking this material as the gold standard for cell sheet production, several works have been proposed with two main purposes: to establish a different temperature to allow the detachment of the cell sheet or to functionalize the surface of the thermoresponsive material to guide cells better into the desired phenotype. Previous works have reported that the temperature at which the transition takes place can be modulated by the introduction of certain copolymers or specific domains that can alter the transition temperature [64]. In this sense, in previous works, PNiPAAm was paired with poly(vinyl methyl ether)-based polymer, allowing not only the change of temperature switch but also provided thickness, stiffness and swelling behavior. CEC were seeded on the surface followed by the detachment of the cell sheet, showing specific CEC markers such as ZO-1 and Na^+^/K^+^-ATPase functionalities before and after the transfer [64, 65]. The combination with other polymers has also been shown to be possible, such as poly(N-N′-(dimethylacrylamide)) [65].

Collagen has generally been used as a biomaterial to allow the culture of CEC on top while allowing its further extraction as a cell layer [66]. The combination with other polymers such as diethyleneglycol methacrylate allowed tuning the temperature for detachment at around 32°C [67]. These cell sheets can be then implanted in rabbit corneas that have previously been denuded of the endothelium, showing that the clarity was gradually increased in the cell sheet group compared to the control groups [68]. In some cases, temperature could decrease up to 20°C and still allowed the detachment of the cell layer with no adverse behavior [69]. An important milestone of these thermo-responsive substrates is to culture them under serum-free conditions to allow its clinical translation [70, 71]. In this sense, thermo-responsive polymers were used with different ECM coatings to allow cell attachment. Interestingly, the type of CEC used had a significant effect on cell behavior, showing that immortalized heterogeneous CEC were less prone to attachment than an immortalized clonally grown CEC line (HCEC-B4G12). It was shown that this cell line was able to produce high levels of vinculin, which allowed having a strong adherence on the substrate even in the absence of serum and without previous conditioning with ECM molecules [70, 71].

Nanotechnology has as well emerged as a promising tool to allow the implantation of cells into the site of defect without the need of a scaffold. In this sense, human umbilical cord blood endothelial progenitor cells were labeled with CD34 immunomagnetic NP. These NP can be strongly attracted by a magnet. The combination of cells labeled with NP allowed to recapitulate the cells with NP and repair the corneal endothelium defects by simply placing the magnet in the anterior chamber [72]. Positive results have been obtained in terms of allowing substituting the endothelium of rabbits, maintaining good levels of intraocular pressure without any signs of corneal edema [73].

## Substrates for CEC implantation

**Figure 7.**
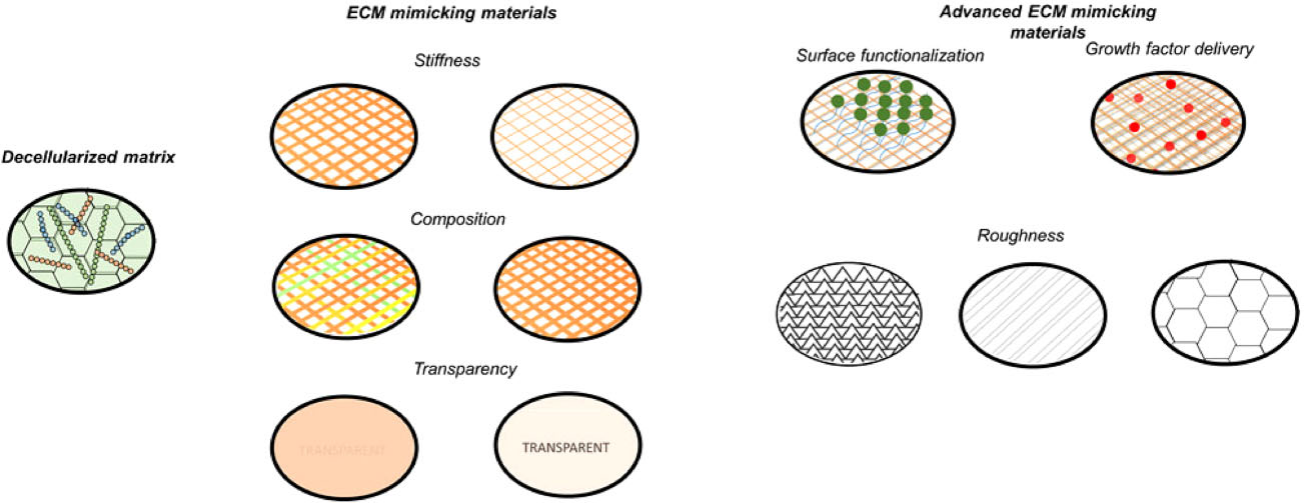
Matrices for enhancing cell behavior. Cells can be cultured in decellularized matrices, in ECM mimicking materials or in advanced ECM mimicking materials.

Besides the previously described cell sheet production, CEC implantation has been ameliorated with the use of several matrixes that can provide a proper environment for cell attachment, proliferation and differentiation. Furthermore, these biomaterials themselves are also able to elicit a positive response in the integration and regeneration of the surrounding tissue. For this purpose, different matrixes with different complexities have been used (Fig. 7). On the one side, decellularized matrixes, which take advantage of a decellularized DM that is able to maintain the native architecture of the CEC environment. On the other side, the use of synthetic matrixes based on biomaterials, like polyvinylidene fluoride (PVDF) or polyvinyl alcohol (PVA), has been increasingly used in CEC implantation [74, 75]. Initially, most matrixes were designed as partially inert allowing only the placement of cells, considered as ECM mimicking matrixes, although their designs have evolved to present higher functionality, giving rise to the advanced mimicking ECM biomaterials. In this section, different examples of the current material used will be described.

### Decellularized matrixes

Commonly used materials in tissue engineering are the decellularized tissues. These types of tissues take advantage of tissues from cadaveric origin or from other animal species, submitting the tissues to several processes to remove the cellular content while maintaining the architecture of the native structure. These have shown adequate results for various tissues, demonstrating that these can be used as proper scaffolds for cell culture, allowing cell proliferation and maintaining the desired phenotype [76]. The corneal endothelium has as well allowed such success, taking advantage of animal corneas, mainly from bovine origin. The posterior lamellae of the cornea can be decellularized by established protocols involving several processing using enzymes as well as detergents. CEC can then be seeded on the DM allowing the formation of a CEC polygonal monolayer, showing positive staining for typical CEC markers [77]. These structures can then be tuned depending on the needs and on different methodologies to improve CEC behavior. In this sense, a bilayered structure was built using an acellular porcine cornea matrix, which already contained good optical clarity, enough toughness for surgical procedures needed, as well as the compatibility needed for tissue engineering [78]. For this purpose, a full thickness cornea substitute was fabricated combining limbal epithelial cell-like cells with CEC. Cells were cultured on each side of the scaffold, creating 3–4 layers of the epithelium and a uniform monolayer of CEC, showing similar densities to native tissues. Immunofluorescence showed that CEC were able to express N-Cadherin, ZO-1 and Na^+^/K^+^-ATPase, which was then correlated with a gradual increase in transparency after an 8-week follow-up postimplantation in rabbits [78]. Similarly, other work used the human anterior lens capsule extracted during cataract surgery, which was then exposed to enzyme digestion and allowed the seeding of CEC on top of it [79]. The results showed that CEC grew in confluence similar to that of native tissue, showing strong positive staining for ZO-1, Connexin-43 and Na^+^/K^+^-ATPase. Hence, this was considered as another plausible scaffold for the *ex vivo* expansion of CEC which will ultimately allow maintaining intact the barrier function and the ionic pump functions [79].

The use of xenogeneic substrates is a plausible approach for corneal endothelium regeneration, although their long-term effects and the presence of remaining cellular residues from the original donor bring controversy on their real clinical use [80]. In order to avoid the use of xenografts, allografts have as well been proposed as substrates for decellularization. In this sense, taking advantage of the discarded corneas of eye donors, these could be digested by enzymatic digestion, allowing the culture of CEC for 14 days. Results showed that cells were functional having a new source of high-quality corneal tissue for transplantation [80]. The main benefit is the presence of ECM-based proteins that are able to induce the binding of CEC to a proper substrate. It was previously described that the use of COL I, COL IV and fibronectin (FN) allowed enhanced adhesion, whereas laminin did not seem to enhance in such manner the cell adhesion and proliferation. For these reasons, providing environments similar to those of native tissues, either using native decellularized tissues or combinations of different biomaterials is essential for a proper regeneration [81]. Collagen has been found as one of the molecules that has enhanced CEC behavior and should hence be considered as a substrate for CEC proliferation and differentiation [82]. Due to the great similarity of other membranes, previous studies also used amniotic membranes as possible carriers for CEC culture and transplant showing positive results with similar results to that of the control [38, 83].

### ECM mimicking biomaterials

Initially, materials based on common polymeric materials were used to see the effect of the material composition on CEC behavior. These type of polymers do not fully resemble the natural ECM, although can be easily functionalized on the surface to endow cues that might be positive for CEC culture. For instance, polymers such as Poly (DL-lactide-co-glycolide) (PDLGA), which is a combination of PLA and glycolic acid, have been previously shown to have beneficial effect on CEC [73]. Interestingly, as the amount of PLA content increased in the samples, the biocompatibility of the membranes decreased, which was probably related with a higher degradation rate and hence a higher acidic pH in the culture media which eventually reduced cell viability [73]. In general, previous studies have tested different synthetic polymers with different degree of hydrophobic properties. In this sense, PVA, poly(ethylene-co-vinyl alcohol), tissue culture polystyrene (TCPS) and PVDF were tested using CEC [73, 74]. It was found that relatively low hydrophobic polymers, such as PVA, inhibited CEC attachment, whereas the more hydrophobic polymers allowed its attachment. Lower hydrophobic polymers showed the tendency to have rather fibroblastic cell morphologies after 3 weeks of culture, whereas the most hydrophobic substrate, PVDF, showed the maintenance of typical hexagonal shape, together with higher expression of CEC-related markers. Interestingly, it was hypothesized that the degree of hydrophobicity found in PVDF enhanced cell-material interaction, stimulating cells to deposit COL IV, establishing a favorable microenvironment for CEC to proliferate and differentiate [74].

Different combinations of synthetic polymers with natural polymers have been performed. For instance, biodegradable polycaprolactone (PCL) substrates and natural polymeric materials such as chitosan were tested. Interestingly, CEC could not attach and proliferate on chitosan, whereas some degree of attachment was found on PCL [84, 85]. For this purpose, combinations of blends were prepared to analyze the effect of combining a natural polymer with a synthetic one [84]. Interestingly, despite PCL has little sites for cell attachment, as the PCL content was increased, the degree of cell adhesion and proliferation increased. Not only did the proliferation increase, but so did the markers of functionality, mainly N-Cadherins and ZO-1, combined with an hexagonal morphology [85]. The enhanced behavior was ascribed to several factors related with the biomaterials, mainly the composition and the topography. In this sense, the blend presented a higher topography than the chitosan by itself as well as the presence of the carbon-oxygen (C=O) bond of PCL, which resulted in higher protein expression of specific markers in the membrane compared to the TCPS, providing a favorable environment in terms of ECM for the culture of CEC [84, 86, 87].

**Figure 8.**
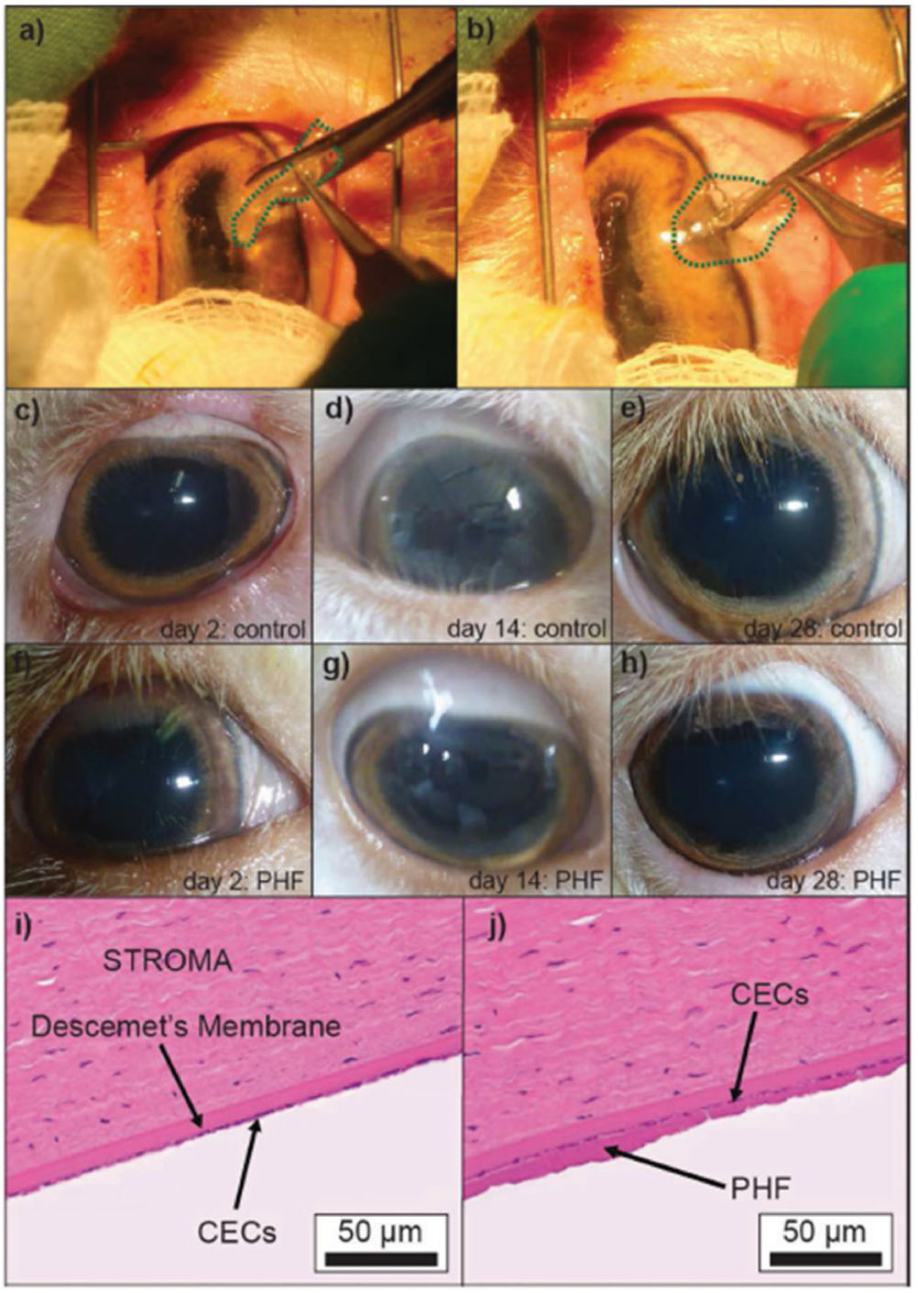
Procedure performed on live sheep corneas (**a**) before and (**b**) after insertion of the poly(ethylene glycol) hydrogel film (PHF) into anterior chamber. Images from (**c–h**) show evolution of transparency of control and implanted PHF, together with H&E staining of (**i**) control and (**j**) PHF after 28 d implantation. Adapted with permission from Ref. [89].

Another promising synthetic polymer that has shown excellent transparency is poly(ethylene glycol) (PEG). This polymer can be manipulated to obtain 50 µm thin films with similar tensile strengths to that of human corneal tissue and with transparency higher than 98% [88, 89]. Furthermore, these types of substrates allowed the proliferation of CEC obtaining a 100% confluent layer after 7 days, allowing its implantation into a sheep corneal model (Fig. 8). Despite the results showed positive results, further biological activity of the biodegradable systems should be sought in order to allow a better cell-material interaction [88, 89]. For this purpose, other combinations of synthetic polymers with natural polymers have combined PLA and cross-linkable gelatin [90]. For this combination, a spin coating technique was used, where droplets of the samples are placed on a rotating device which is then spined to allow centrifugal forces to create a thin coating. For this purpose, poly(d, L-lactide-block-acrylic acid) and different types of gelatin were used to form multilayered structures to allow the culture of CEC. The technique allowed the formation of ultra-thin layers (lower than 1 µm) with high transparency (over 90%) and even showing similar behavior to control in term of *in vitro* behaviors. Hence, this methodology shows a plausible substitute for natural corneas [90]. Although synthetic polymers and composites are having promising results, *in vivo* studies are still required.

Rather than using synthetic grafts, some researchers have taken advantage of natural polymers such as collagen. Collagen has been shown to have adequate behavior when CEC were cultured and these were subsequently transplanted [39]. Other natural polymers such as hyaluronic acid have as well been used for corneal endothelium as a strategy to mimic the native tissue due to their presence in natural endothelium [91]. Nevertheless, collagen has been generally combined with other polymers to allow maintaining stability, since their degradation is generally fast. In this sense, the formation of interpenetrating networks with other polymers such as chitosan or sodium hyaluronate have been previously used, showing good transparency and excellent behavior of CEC seeded on top of the substrates. Similarly, chitosan has been combined as well with gelatin and chondroitin sulfate [92–94]. Despite its degradation can be observed after 5 months of implantation, the results are promising to continue exploring these blends of materials [95, 96]. Interestingly, despite results combining gelatin and chitosan have shown adequate results, blending gelatin with chitin has shown enhanced properties, presenting milder immune response although with a faster degradation, having full degradation in <8 weeks [97]. Chitosan was previously cross-linked with glutaraldehyde to increase the degradation rates and the mechanical properties [98]. For this purpose, collagen vitrigel, which is mainly a collagen sheet with transparent properties, was obtained using porcine atelocollagen and UV irradiation, allowing the culture of CEC on the surface. The vitrigel was given a spherical morphology to mimic to a higher extent the native tissue, showing that their consistency was better compared to the use of flat sheets once implanted in an animal model [99]. The vitrigel membrane together with CEC were transplanted into a rabbit model, showing that after 14 days, the CEC completely covered the grafts after this period of time, expressing high levels of ZO-1 and Na^+^/K^+^-ATPase. These results were significantly better than the control groups in the absence of transplanted CEC [100]. Similar to vitrigel, other strategies based on collagen have prepared membranes by compressing collagen hydrogel until a thin layer is formed, showing as well excellent characteristic of the seeded cells on top. Nevertheless, the transparency of the membrane still remains a further issue to assess [101]. In order to overcome issues with transparency, gelatin, which is denatured collagen, can be easily processed, which upon mixing with heparin, allows an excellent tuning. In this sense, the mechanical properties could be increased by adjusting the heparin ratio as well as the crosslinking of the gelatin or using dehydrothermal processing, as well as changing the molecular weight and the properties of the gelatin [102–105]. Similarly, the mechanical properties of the compressed collagen can as well be improved by combining the sheets with PLGA aligned nanofibers [106]. Furthermore, collagen has been shown to be still distant from the gold standard, which is the use of human anterior lens capsules. These membranes were compared with bioengineered collagen sheets of different thickness, mainly 20 and 100 µm. The results showed that the collagen membranes behaved in an adequate manner but were still distant from the optimum control. The membranes were either too soft for the proper placement into the site of defect or too rigid to allow introducing the membranes within the keratoplasty. Nevertheless, the culture of CEC *in vitro* behaved in an excellent manner, showing proper morphology and adequate phenotype expression [107]. Hence, for further clinical applications, these materials still require improving the mechanical properties for optimal implantation.

### Advanced CEC mimicking biomaterials

#### Molecule eluting biomaterials

Cells are able to communicate among them through the release of molecules that are sent as signaling cues. These molecules are able to elicit changes in the phenotypes of the receiving cells, affecting their phenotype and ultimately activating regeneration pathways. Taking into account that most of the materials used in CEC tissue engineering are based on water-soluble polymers, the previously mentioned molecules can be entrapped within these matrixes to allow a sustained release over time and simulate the desired phenotypes and regeneration pathways. The excellent water adsorption capacity of gelatin and the fine-tuning when combined with heparin allowed the fabrication of membranes that encapsulated growth factors, as bFGF that was shown to increase the proliferative capacity of cells, and were able to release them in a sustained manner. Most importantly, cells cultured on the membranes presented adequate surface markers for CEC and had enough rigidity for manipulation and implantation [104]. Hence, this provides evidence on the use of drug-eluting biomaterial implanted in the cornea rather than the direct placement of cell sheets or cell-loaded scaffolds [104]. Related with the release of molecules, dorzolamide hydrochloride was coated on the surface of PCL-based materials to release it once implanted in rabbit eyes, showing a decrease on the intraocular pressure [108, 109]. Furthermore, these materials were shown to be biocompatible with CEC, although no understating of cellular markers was given. Optimization of the membrane should be performed in order to obtain near-order kinetics rather than burst release. This could be mitigated by actually incorporating the drugs within the scaffolds rather than performing coatings [110]. Similar study was performed using gelatin cross-linked with a carbodiimide, which depending on the degree of crosslinking, allowed the release of the loaded drug within the initial 8 h [110]. Once again, the soaking of the drug within the surface reduces the sustained delivery and hence strategies to provide a prolonged release are needed. In a more sophisticated system, microspheres made of biodegradable PLGA incorporating ROCK inhibitor Y-27632 were prepared using gelatin in a double emulsion, which were injected into the anterior chamber in rabbit eyes [111]. ROCK inhibitor has been proposed as a treatment in clinical scenario, although patients still require the use of immunosuppressive treatments during a long period of time to avoid any immune response [112]. The release of the ROCK inhibitor allowed the enhancement of CEC proliferation *in vitro* and the release rate could be controlled by tuning the amount of lactic and glycolic ratio. Despite of the positive results *in vitro*, the release was shown to be complete after 10 days at most, which limits further outcomes of the described microspheres [111, 112]. Hence, other encapsulation strategies that allow a longer release must be sought as well as strategies to increase the sustained release of drugs.

#### Surface modification

Similar to drug-eluting materials, materials that are able to modulate the cell-material interaction as well as the tissue-material interaction by modifying the surface properties have as well gained significant attention in endothelium regeneration. In this sense, two commonly used strategies are based on the exposure of functionalized surfaces for cells to interact with, or the use of substrates with topographical cues. Some strategies have involved sophisticated technologies for performing topographies or the surface functionalization, whereas others are based on simple material mixtures. In the latter one, some strategies have used combinations of synthetic and natural polymers, such as blends of glycerol and silk fibroin. Glycerol may have great potential due to the transparent properties, but lacks of enough mechanical stability as well as limited biological interactions. In a similar manner, silk fibroin by itself lacks of enough transparency although has excellent biocompatibility and mechanical properties [113]. Nevertheless, during the preparation process cracks may appear, which have been shown to decrease in the presence of glycerol [114]. Hence, their combination has shown a rather uniform structure, with a reduced thickness and a rougher surface. The results demonstrated that CEC were shown to proliferate better on surfaces that had intermediate roughness with the presence of 1% glycerol in silk fibroin. The need of slight roughness for enhancing the behavior of cells is based on the improvement of protein adsorption on the surface of the substrate which inherently enhanced CEC interaction with the substrate [115].

**Figure 9.**
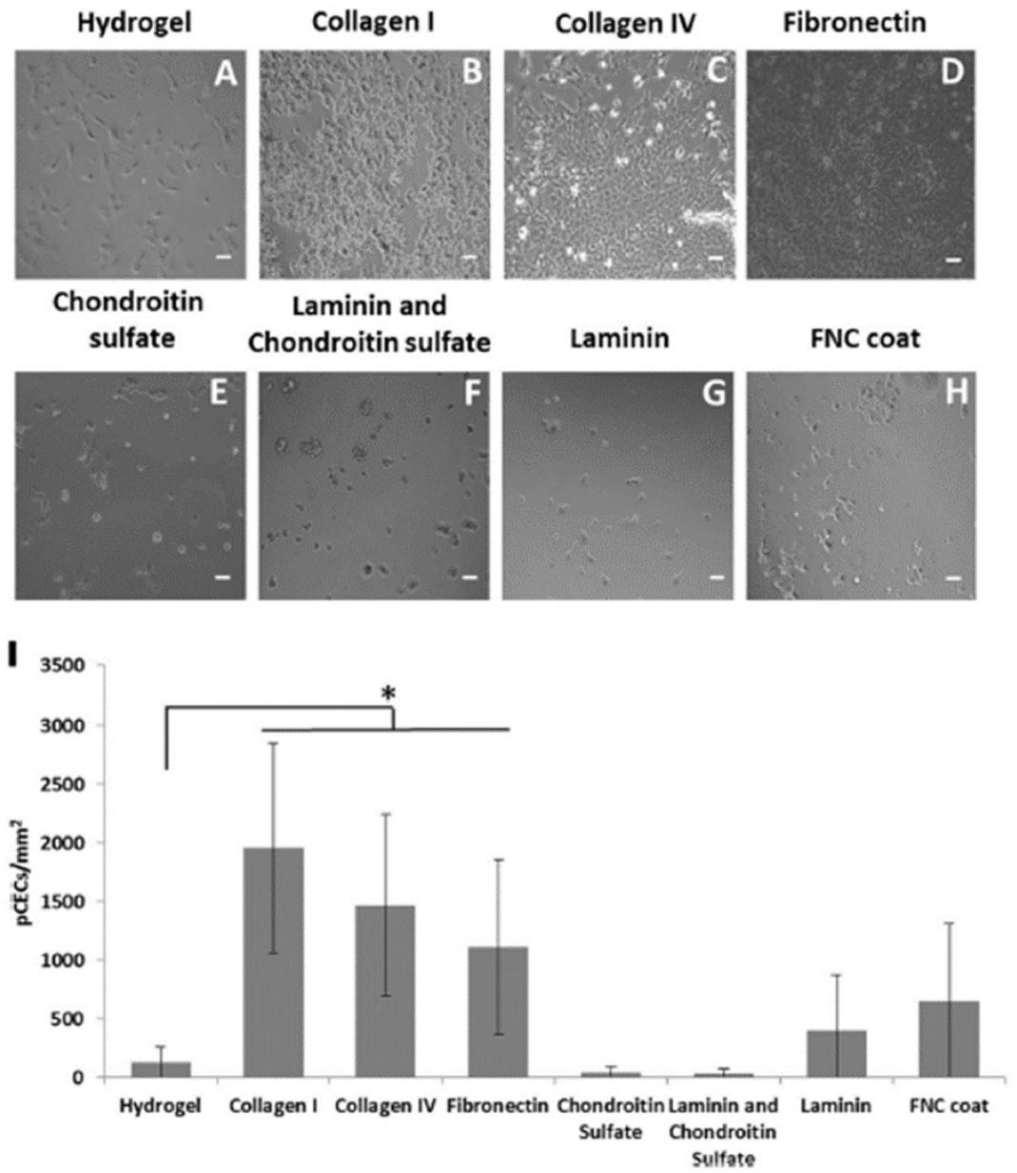
Representative images of adhesion of CEC on poly-ε-lysine hydrogel uncoated or combined with the different proteins. Symbol * denotes significantly different *P* < 0.05 compared to poly-ε-lysine hydrogel only (**A–H**). The images were quantified to clearly observe the differences in cell adhesion at 24 h (**I**). Scale bar 100 μm. Adapted with permission from Ref. [117].

Other strategies have coated silk fibroin with certain molecules such as COL IV, FN and chondroitin sulfate-laminin mixture, showing that the optimum results for fibroin treatment were collagen [82]. Agarose has as well been functionalized with different peptides to enhance the biological properties, showing that in general, gelatin-derived signals showed the best behavior of cultured CEC [116]. Similar studies were performed with Poly lysine as base substrate, which could as well be modified with synthetic peptides, such as RGD, to promote CEC behavior. Furthermore, their combination with natural-based proteins showed enhanced cell attachment and enhanced functionality (Fig. 9) [117]. In a similar way, beta-carotenoids have as well been placed on the surface of silk fibroin to enhance the proliferation and enhancement of phenotypic response, as it is known that beta-carotenoids are able to protect patients suffering from eye diseases, due to its anti-oxidant properties [118]. The results showed an enhancement compared to the pristine silk film, mainly related to the proliferation and to the expression of CEC markers [118]. In an attempt to further increase the properties of silk, lysophosphatidic acid (LPA) was incorporated within the structure of silk fibroin. LPA is an endogenous glycerophospholipid signaling molecule, which was previously shown to stimulate growth of different cell types as well as enhancing several biological functionalities. The results showed that the physicochemical properties were generally unaltered. Contrary to this, the biological properties of the composite containing LPA compared to pristine silk had enhanced biological functionalities, showing higher biocompatibility and higher specific markers expression [119].

In a similar manner, other natural polymers, such as gelatin, have been combined with synthetic ones to achieve enhanced properties. Nevertheless, not only is the composition important but also the morphology of the matrix is of even more relevance. The morphology of the substrates can be controlled at the macro or at the micron level. At the macroscopic level, it is relatively affordable and cost-effective to produce material sheets with optimized curvatures to mimic the curvature at which natural endothelium can be found [120]. For this purpose, a curved gelatin hydrogel sheet was prepared by hydrothermal method allowing the culture of CEC as well as its implantation in monkeys with bullous keratopathy. Interestingly, the sheets showed similar permeability to water and protein as controls, mainly atelocollagen and vitrigel sheets. An importance of the curved sheets is that there was no wrinkling of the structure after implantation, whereas the flat sheets produced wrinkles which inhibited proper cell function [120]. Moreover, patterned structures at the micro and nano level that can be created during the processing of the materials is an interesting approach to control the CEC fate [121]. For instance, gelatin methacrylate has been used to create patterned structures, showing that rather than the composition, the presence of gratings with different heights and widths had a direct effect on CEC behavior. More specifically, the gratings with 1 µm pillars with square and hexagonal morphology were optimum [121]. Despite the presence of macro structural features are interesting to modulate and conform CEC in a hexagonal manner, the morphology of the matrixes at the nano level may as well have profound effect on CEC behavior. For instance, combinations of poly (glycerol sebacate) (PGS)-PCL have been combined in the form of nanofibrous structures formed by electrospinning [122]. Cultured CEC on the nanofibrous membranes allowed the formation of monolayers with the hexagonal morphology. Interestingly, increasing the ratios of PGS/PCL showed greater results in terms of organization and functionality [123]. Similarly, poly(methyl-metacrylate) (PMMA), PLGA and PCL were as well used by electrospinning. The results showed differences in terms of nanofiber size, having greater sizes for PMMA and PCL nanofibers than for the PLGA. Interestingly, only the PLGA preserved a normal hexagonal CEC morphology together with a higher toxicity for the PMMA nanofibers. Taken together, PLGA nanofibers have shown to be optimal compared to PCL and PMMA nanomatrixes and hence have certain potential in corneal endothelium replacement [53]. Nevertheless, despite of the positive cell–matrix interaction, further studies are needed in terms of transparency, adhesion and *in vivo* behavior. For this purpose, combinations of synthetic polymers with silk fibroin were shown to have promising results. It was observed that the use of small amounts of silk fibroin within the structure of PLA-PCL copolymers significantly increased the transparency of the matrixes. Furthermore, it was shown that the incorporation of silk fibroin: PCL–PLA in a weight ratio of 25:75 showed highest proliferation of CEC [124]. It was previously demonstrated that by simply controlling the topography, without the addition of any biochemical signaling, the CEC fate and proliferation could be controlled in such as similar way. In this sense, several micro well and pits were fabricated showing that 1 µm pillars could enhance proliferation as well as ZO-1 expression, showing in general that topography is mandatory for the culture of CEC [125].

**Figure 10.**
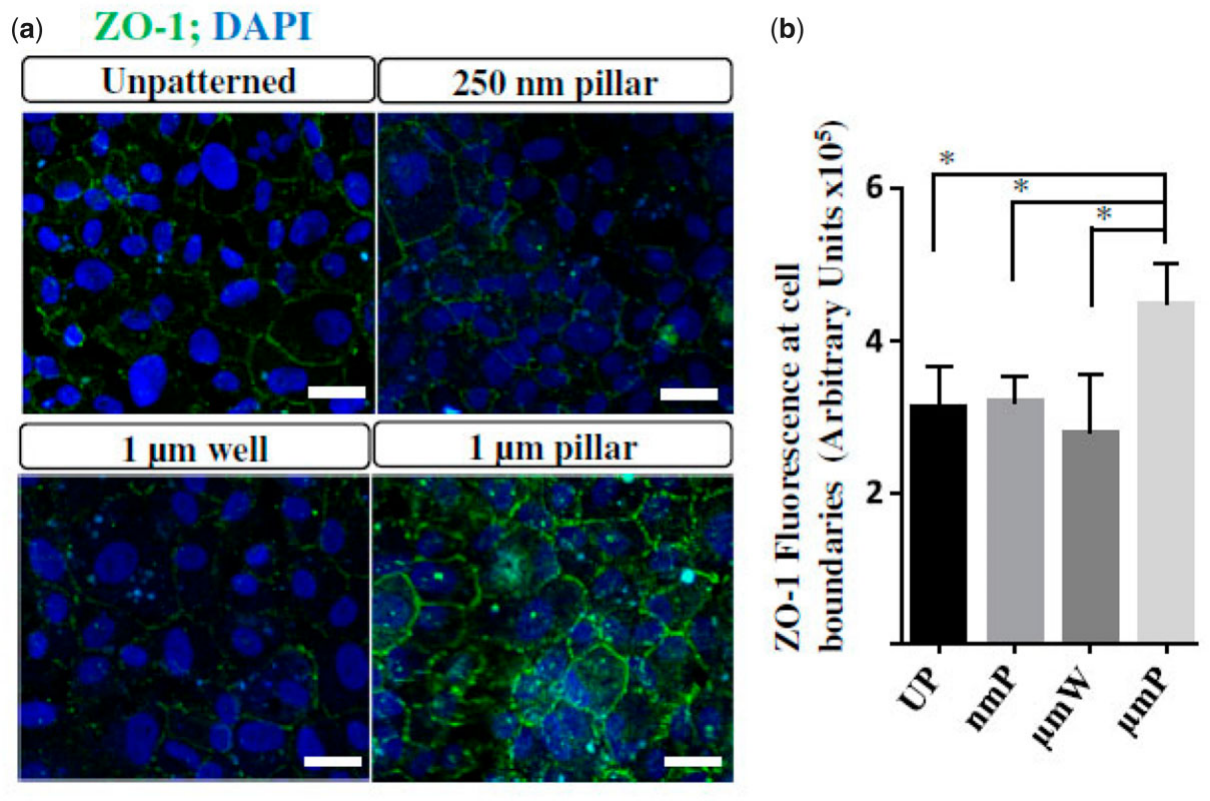
Confocal microscopy images of immunofluorescence staining (**A**) and quantification (**B**) of CEC tight junction protein (ZO-1) on fibronectin and collagen mix (FNC) coated patterned substrates at 7 days (scale bar = 50 µm). Adapted with permission from Ref. [125].

Since the topographical effect is inherently linked with a proper adhesion onto the substrate, topographical cues were combined with chemical cues, providing microwells and micropillars with different coatings on PDMS substrates [125, 126]. Interestingly, the results showed that not only the topographical cues are important but also the chemical cues, showing that optimum results were obtained when micropillar coated with FN and collagen mix were placed, observing highest CEC gene expression (Fig. 10). Nevertheless, further studies are needed to assess the trend using different patterns, as the results were not completely conclusive [127]. Furthermore, these studies have been performed in non-implantable materials, such as Polydimethylsiloxane (PDMS), so efforts should strive in obtaining similar results in implantable materials, such as collagen, gelatin or synthetic polymers. That is why a more applied technology using titanium as substrate, which is the same titanium used in the keratoprosthesis, showed that there was no effect on the range of topographies used, having a maximum Ra of 1.15 µm [128].

Even the use of short peptides that are able to self-assemble in aqueous media have been proposed for the encapsulation of CEC [129]. Actually, one of the main limitations of self-assembling peptides is the limited manipulation ability, which make them less viable for clinical applications. The short peptides based on Isoleucine-Valine-Lysine-Cysteine (IVKC) allowed the encapsulation and survival of CEC.

## Conclusions and future perspectives

Current strategies to develop new materials from the successfully CEC transplant have been explored. The technologies previously described address two main strategies, mainly the use of cell sheets, where biomaterials are used to allow the initial attachment and proliferation to subsequently detach the cell sheet, whereas another strategy takes into account the use of biomaterials as implantable substrates for tissue engineering.

All these technologies are still in an emergent status in the field of corneal endothelium, but is a promising strategy and valuable in other tissues, reaching clinical scenarios and showing promising results, even having established several companies in the field. In this sense, a significant number of companies have appeared in the last decade involving the use of biomaterials and their combinations for the regeneration of tissues [130]. In this sense, skin has for instance been tissue engineered for the production of this synthetic graft for patients with burns and similar situations, providing, in combination with autologous cells in some cases, a great combination for the faster regeneration of burned patients [131, 132]. In a similar way, cartilage tissue regeneration has been established as well combining collagen-based biomaterials with chondrocyte-like cells, which have been acquired from different cell sources, having mesenchymal stem cells (MSC) as a main source [133, 134]. As a final example, nerve regeneration, which is an extremely complex tissue, has proven the use of different cell guiding biomaterials with specific microstructures combined with different type of growth factors, such as neural growth factor, together with cells has provided optimistic results [135–137].

Cell sheets are generally based on different methodologies to control the stem cell fate or to control the ability of CEC to maintain the adequate phenotype, mainly using chemical substrates that can modulate the stem cell fate. The control over the stem cell fate has generally depended on the use of genes as well as chemical substrates that may limit further applications in real clinical scenarios. The use of cells extracted from the same patient that are cultured on substrates that allow the fabrication of cell sheets has been shown as an excellent approach for autologous transplantation. The role of stem cells and how to obtain autologous cells from the patients should be the direction in which research should be striving. These substrates have to be meticulously designed to allow controlling the appropriate detachment of cells in order to avoid affecting the cell sheet and the properties of the non-functional cells.

In a similar way, biomaterials need to be enhanced as the ideal partner for tissue engineering of the cornea. These biomaterials need to be designed and systematically studied in terms of topography, stiffness, curvatures, chemical composition or surface charge among others, which will provide physicochemical elements necessary to control the phenotype of the cultured CEC. Despite some works have shown their potential, results have been sparse and not placed together to understand the needed properties of fully functional scaffolds. Other scaffolds substrates for the adequate regeneration of the corneal endothelium such as nanofibers or NP, as well the use of drug delivery systems should be enhanced. Nanotechnology has arisen as a methodology to carefully allow directing cell behavior, which combined with drug delivery systems that elute specific amounts of signaling molecules in short periods of time can have great benefit. The eluted molecules may modulate an antibacterial response, reduce an inflammatory response and even enhance the cell response of the CEC present. Finally, new technologies such as 3D printing should be taken into account to further produce biomaterials that can be patient specific.

In this sense, rather than the use of substrates onto which cells can be cultured, injectable hydrogels have gained great interest in the last years. These can be controlled and tuned according to its chemistry and can be cross-linked in several ways, mainly physical stimuli, magnetic stimuli or chemical stimuli. Injectable hydrogels offer a non-invasive system to allow the *in situ* placement of materials, which can be combined with cells or drugs to enhance the tissue regeneration. In this sense, several approaches have been made for this purpose. The use of hydrogels opens the route of cell encapsulation using hydrogels to construct patient-specific biomaterials with biological functionality using 3D printing [138]. For instance, chitosan, hydroxypropil chitosan and sodium alginate were combined to allow the encapsulation of CEC which were then placed on a DM [139]. The CEC were able to survive as well as maintained a normal morphology, which opens a new opportunity for corneal endothelium reconstruction by *in situ* hydrogel formation [140]. Similarly, oxidized dextran cross-linked with adipic acid dihydrazide hydrogels were used as injectable systems. Interestingly, the time of gelation as well as their degradation properties could be controlled by tuning the degree of dextran oxidation and the concentration of both components. Results showed that cell adhesion and proliferation could easily take place, although low oxidized dextrans showed confluence in just 24 h of culture [140]. Furthermore, the release products from the dextrans did not show cytotoxicity indirect cultures, rendering excellent biocompatibility.

## Funding

This work was funded by the Government of Catalonia (2017 SGR 708), the Spanish Ministry (Ramón y Cajal fellowship (RYC2018-025977-I) and project RTI2018-096088-J-100 (MINECO/FEDER)), and predoctoral fellowship from Generalitat de Catalunya (FI).

*Conflicts of interest statement.* None declared.

## References

[1] Mathews PM , Lindsley K, Aldave AJ, Akpek EK. Etiology of global corneal blindness and current practices of corneal transplantation: a focused review. Cornea 2018;37:1198–6. 29912039 10.1097/ICO.0000000000001666

[2] Whitcher JP , Srinivasan M, Upadhyay MP. Corneal blindness: a global perspective. Bull World Health Organ 2001;79:214–21. 11285665 PMC2566379

[3] Gain P , Jullienne R, He Z, Aldossary M, Acquart S, Cognasse F, Thuret G. Global survey of corneal transplantation and eye banking. JAMA Ophthalmol 2016;134:167–73. 26633035 10.1001/jamaophthalmol.2015.4776

[4] Boynton GE , Woodward MA. Evolving techniques in corneal transplantation. Curr Surg Reports 2015;3. doi:10.1007/s40137-014-0079-5. PMC4474142 26101726

[5] Eye Bank Association of America, n.d. https://restoresight.org/members/publications/statistical-report/

[6] Navaratnam J , Utheim TP, Rajasekhar VK, Shahdadfar A. Substrates for expansion of corneal endothelial cells towards bioengineering of human corneal endothelium. J Funct Biomater 2015;6:917–45. 26378588 10.3390/jfb6030917 PMC4598685

[7] Morishige N , Sonoda KH. Bullous keratopathy as a progressive disease: evidence from clinical and laboratory imaging studies. Cornea 2013;32:S77–S83. 24104939 10.1097/ICO.0b013e3182a1bc65

[8] Fahmy R , Fahmy RM. Correlation between Corneal Endothelial Cell Characteristics and Dry Eye Disease. Medical & Surgical Ophthalmology Research. 2018;1. doi:10.31031/MSOR.2018.01.000509.

[9] Bourne WM. Biology of the corneal endothelium in health and disease. Eye (Lond) 2003;17:912–8. 14631396 10.1038/sj.eye.6700559

[10] Labelle P. The eye. In: Zachary JF (ed). Pathologic Basis of Veterinary Disease Expert Consult. Mosby, Missouri, USA. 2016, 1265–1318.e1. doi:10.1016/B978-0-323-35775-3.00021-7.

[11] Wörner CH , Olguín A, Ruíz-García JL, Garzón-Jiménez N. Cell pattern in adult human corneal endothelium. PLoS One 2011;6:e19483. 21602935 10.1371/journal.pone.0019483 PMC3094353

[12] Bonanno JA. Molecular mechanisms underlying the corneal endothelial pump. Exp Eye Res 2012;95:2–7. 21693119 10.1016/j.exer.2011.06.004 PMC3199349

[13] Ventura AC , Wälti R, Böhnke M. Corneal thickness and endothelial density before and after cataract surgery. Br J Ophthalmol 2001;85:18–20. 11133705 10.1136/bjo.85.1.18 PMC1723680

[14] Ali M , Raghunathan VK, Li JY, Murphy CJ, Thomasy SM. Biomechanical relationships between the corneal endothelium and Descemet’s membrane. Exp Eye Res 2016;152:57–70. 27639516 10.1016/j.exer.2016.09.004 PMC5370075

[15] Thomasy SM , Raghunathan VK, Winkler M, Reilly CM, Sadeli AR, Russell P, Jester JV, Murphy CJ. Elastic modulus and collagen organization of the rabbit cornea: epithelium to endothelium. Acta Biomater 2014;10:785–91. 24084333 10.1016/j.actbio.2013.09.025 PMC4280096

[16] Gutermuth A , Maassen J, Harnisch E, Kuhlen D, Sauer-Budge A, Skazik-Voogt C, Engelmann K. Descemet’s membrane biomimetic microtopography differentiates human mesenchymal stem cells into corneal Endothelial-Like cells, cornea. Cornea 2019;38:110–9. 30308581 10.1097/ICO.0000000000001765 PMC6282677

[17] Perez RA , Choi S-J, Han C-M, Kim J-J, Shim H, Leong KW, Kim H-W. Biomaterials control of pluripotent stem cell fate for regenerative therapy. Prog Mater Sci 2016;82:234–93.

[18] Pérez RA , Won J-E, Knowles JC, Kim H-W. Naturally and synthetic smart composite biomaterials for tissue regeneration. Adv Drug Deliv Rev 2013;65:471–96. 22465488 10.1016/j.addr.2012.03.009

[19] Rizwan M , Peh GS, Adnan K, Naso SL, Mendez AR, Mehta JS, Yim EKF. In vitro topographical model of Fuchs dystrophy for evaluation of corneal endothelial cell monolayer formation. Adv Healthc Mater 2016;5:2896–910. 27701826 10.1002/adhm.201600848

[20] Jakus MA. Studies on the cornea. II. The fine structure of Descement’s membrane. J Biophys Biochem Cytol 1956;2:243–52. 13357549 10.1083/jcb.2.4.243 PMC2229738

[21] Sawada H , Konomi H, Hirosawa K. Characterization of the collagen in the hexagonal lattice of Descemet’s membrane: its relation to type VIII collagen. J Cell Biol 1990;110:219–27. 2104858 10.1083/jcb.110.1.219 PMC2115983

[22] Danielsen CC. Tensile mechanical and creep properties of Descemet’s membrane and lens capsule. Exp Eye Res 2004;79:343–50. 15336496 10.1016/j.exer.2004.05.014

[23] Yamamoto A , Tanaka H, Toda M, Sotozono C, Hamuro J, Kinoshita S, Ueno M, Tanaka M. A physical biomarker of the quality of cultured corneal endothelial cells and of the long-term prognosis of corneal restoration in patients. Nat Biomed Eng 2019;3:953–60. 31332343 10.1038/s41551-019-0429-9

[24] Vinciguerra P , Roberts CJ, Albé E, Romano MR, Mahmoud A, Trazza S, Vinciguerra R. Corneal curvature gradient map: a new corneal topography map to predict the corneal healing process. J Refract Surg 2014;30:202–7. 24763726 10.3928/1081597X-20140218-02

[25] Paolo Vinciguerra CA. Corneal curvature gradient determines corneal healing process and epithelial behavior. J Refract Surg 2015;31:282–4. 25884584 10.3928/1081597X-20150319-08

[26] Gouveia RM , Koudouna E, Jester J, Figueiredo F, Connon CJ. Template curvature influences cell alignment to create improved human corneal tissue equivalents. Adv Biosys 2017;1:1700135. 10.1002/adbi.201700135 32646159

[27] Kim KW , Park SH, Lee SJ, Kim JC. Ribonuclease 5 facilitates corneal endothelial wound healing via activation of PI3-kinase/Akt pathway. Sci Rep 2016;6:31162. 27526633 10.1038/srep31162 PMC4985649

[28] Grant MB , Khaw PT, Schultz GS, Adams JL, Shimizu RW. Effects of epidermal growth factor, fibroblast growth factor, and transforming growth factor-beta on corneal cell chemotaxis. Invest Ophthalmol Vis Sci 1992;33:3292–301. 1428704

[29] Nayak SK , Binder PS. The growth of endothelium from human corneal rims in tissue culture. Invest Ophthalmol Vis Sci 1984;25:1213–6. 6384123

[30] Hoppenreijs VP , Pels E, Vrensen GF, Oosting J, Treffers WF. Effects of human epidermal growth factor on endothelial wound healing of human corneas. Invest Ophthalmol Vis Sci 1992;33:1946–57. 1582800

[31] Hoppenreijs VP , Pels E, Vrensen GF, Treffers WF. Basic fibroblast growth factor stimulates corneal endothelial cell growth and endothelial wound healing of human corneas. Invest Ophthalmol Vis Sci 1994;35:931–44. 8125756

[32] Woost PG , Jumblatt MM, Eiferman RA, Schultz GS. Growth factors and corneal endothelial cells: I. Stimulation of bovine corneal endothelial cell DNA synthesis by defined growth factors. Cornea 1992;11:1–10. 1559341 10.1097/00003226-199201000-00001

[33] Zhang W , Chen J, Fu Y, Fan X. The signaling pathway involved in the proliferation of corneal endothelial cells. J Recept Signal Transduct Res 2015;35:585–91. 26360823 10.3109/10799893.2015.1026445

[34] Díaz ME , González L, Miquet JG, Martínez CS, Sotelo AI, Bartke A, Turyn D. Growth hormone modulation of EGF-induced PI3K-Akt pathway in mice liver. Cell Signal 2012;24:514–23. 22019461 10.1016/j.cellsig.2011.10.001 PMC3616332

[35] Lee JG , Heur M. WNT10B enhances proliferation through β-Catenin and RAC1 GTPase in human corneal endothelial cells. J Biol Chem 2015;290:26752–64. 26370090 10.1074/jbc.M115.677245 PMC4646328

[36] Sabater AL , Andreu EJ, García-Guzmán M, López T, Abizanda G, Perez VL, Moreno-Montañés J, Prósper F. Combined PI3K/akt and Smad2 activation promotes corneal endothelial cell proliferation. Invest Ophthalmol Vis Sci 2017;58:745–54. 28146239 10.1167/iovs.16-20817

[37] Li W , Sabater AL, Chen Y-T, Hayashida Y, Chen S-Y, He H, Tseng SCG. A novel method of isolation, preservation, and expansion of human corneal endothelial cells. Invest Ophthalmol Vis Sci 2007;48:614–20. 17251457 10.1167/iovs.06-1126 PMC3196988

[38] Ishino Y , Sano Y, Nakamura T, Connon CJ, Rigby H, Fullwood NJ, Kinoshita S. Amniotic membrane as a carrier for cultivated human corneal endothelial cell transplantation. Invest Ophthalmol Vis Sci 2004;45:800–6. 14985293 10.1167/iovs.03-0016

[39] Mimura T , Yamagami S, Yokoo S, Usui T, Tanaka K, Hattori S, Irie S, Miyata K, Araie M, Amano S. Cultured human corneal endothelial cell transplantation with a collagen sheet in a rabbit model. Invest Ophthalmol Vis Sci 2004;45:2992–7. 15326112 10.1167/iovs.03-1174

[40] Chen X , Wu L, Li Z, Dong Y, Pei X, Huang Y, Wang L. Directed differentiation of human corneal endothelial cells from human embryonic stem cells by using cell-conditioned culture media. Invest Ophthalmol Vis Sci 2018;59:3028–36. 30025120 10.1167/iovs.17-23627

[41] Zhang K , Pang K, Wu X. Isolation and transplantation of corneal endothelial cell–like cells derived from in-vitro-differentiated human embryonic stem cells. Stem Cells Dev 2014;23:1340–54. 24499373 10.1089/scd.2013.0510 PMC4046205

[42] Wagoner MD , Bohrer LR, Aldrich BT, Greiner MA, Mullins RF, Worthington KS, Tucker BA, Wiley LA. Feeder-free differentiation of cells exhibiting characteristics of corneal endothelium from human induced pluripotent stem cells. Biol Open 2018;7. doi:10.1242/bio.032102. PMC5992532 29685994

[43] McCabe KL , Kunzevitzky NJ, Chiswell BP, Xia X, Goldberg JL, Lanza R. Efficient generation of human embryonic stem cell-derived corneal endothelial cells by directed differentiation. PLoS One 2015;10:e0145266. 26689688 10.1371/journal.pone.0145266 PMC4686926

[44] Hatou S , Sayano T, Higa K, Inagaki E, Okano Y, Sato Y, Okano H, Tsubota K, Shimmura S. Transplantation of iPSC-derived corneal endothelial substitutes in a monkey corneal edema model. Stem Cell Res 2021;55:102497. 34411973 10.1016/j.scr.2021.102497

[45] Shao C , Fu Y, Lu W, Fan X. Bone marrow-derived endothelial progenitor cells: a promising therapeutic alternative for corneal endothelial dysfunction. Cells Tissues Organs 2011;193:253–63. 20962503 10.1159/000319797

[46] Hatou S , Yoshida S, Higa K, Miyashita H, Inagaki E, Okano H, Tsubota K, Shimmura S. Functional corneal endothelium derived from corneal stroma stem cells of neural crest origin by retinoic acid and Wnt/b-Catenin signaling. Stem Cells Dev 2013;22:828–39. 22974347 10.1089/scd.2012.0286

[47] Dai Y , Guo Y, Wang C, Liu Q, Yang Y, Li S, Guo X, Lian R, Yu R, Liu H, Chen J. Non-genetic direct reprogramming and biomimetic platforms in a preliminary study for adipose-derived stem cells into corneal endothelia-like cells. PLoS One 2014;9:e109856. 25333522 10.1371/journal.pone.0109856 PMC4198143

[48] Inagaki E , Hatou S, Higa K, Yoshida S, Shibata S, Okano H, Tsubota K, Shimmura S. Skin-derived precursors as a source of progenitors for corneal endothelial regeneration. Stem Cells Transl Med 2017;6:788–98. 28186681 10.1002/sctm.16-0162 PMC5442762

[49] Yamashita K , Inagaki E, Hatou S, Higa K, Ogawa A, Miyashita H, Tsubota K, Shimmura S. Corneal endothelial regeneration using mesenchymal stem cell derived from human umbilical cord. Stem Cells Dev 2018;27:1097–108. doi:10.1089/scd.2017.0297. 29929442

[50] Bosch BM , Salero E, Núñez-Toldrà R, Sabater AL, Gil FJ, Perez RA. Discovering the potential of dental pulp stem cells for corneal endothelial cell production: a proof of concept. Front Bioeng Biotechnol 2021;9:617724. 33585434 10.3389/fbioe.2021.617724 PMC7876244

[51] Perez RA , Jung CR, Kim HW. Biomaterials and culture technologies for regenerative therapy of liver tissue. Adv Healthcare Mater 2017;6:1600791. 10.1002/adhm.201600791 27860372

[52] Lee EJ , Kasper FK, Mikos AG. Biomaterials for tissue engineering. Ann Biomed Eng 2014;42:323–37. 23820768 10.1007/s10439-013-0859-6 PMC3844045

[53] Kruse M , Walter P, Bauer B, Rütten S, Schaefer K, Plange N, Gries T, Jockenhoevel S, Fuest M. Electro-spun membranes as scaffolds for human corneal endothelial cells. Curr Eye Res 2018;43:1–11. 29281419 10.1080/02713683.2017.1377258

[54] Mimura T , Yamagami S, Amano S. Corneal endothelial regeneration and tissue engineering. Prog Retin Eye Res 2013;35:1–17. 23353595 10.1016/j.preteyeres.2013.01.003

[55] Kim YJ , Lim H, Li Z, Oh Y, Kovlyagina I, Choi IY, Dong X, Lee G. Generation of multipotent induced neural crest by direct reprogramming of human postnatal fibroblasts with a single transcription factor. Cell Stem Cell 2014;15:497–506. doi:10.1016/J.STEM.2014.07.013. 25158936

[56] Rana D , Zreiqat H, Benkirane-Jessel N, Ramakrishna S, Ramalingam M. Development of decellularized scaffolds for stem cell-driven tissue engineering. J Tissue Eng Regen Med 2017;11:942–65. 26119160 10.1002/term.2061

[57] Xue J , Wu T, Dai Y, Xia Y. Electrospinning and electrospun nanofibers: methods, materials, and applications. Chem Rev 2019;119:5298–415. 30916938 10.1021/acs.chemrev.8b00593 PMC6589095

[58] Lee JH. Injectable hydrogels delivering therapeutic agents for disease treatment and tissue engineering. Biomater Res 2018;22:1–14. 30275970 10.1186/s40824-018-0138-6 PMC6158836

[59] Sun Y , Nan D, Jin H, Qu X. Recent advances of injectable hydrogels for drug delivery and tissue engineering applications. Polym Test 2020;81:106283.

[60] Berkland C , Kim K, Pack DW. PLG microsphere size controls drug release rate through several competing factors. Pharm Res 2003;20:1055–62. 12880292 10.1023/a:1024466407849

[61] Zhao J , Stenzel MH. Entry of nanoparticles into cells: the importance of nanoparticle properties. Polym Chem 2018;9:259–72.

[62] Nagase K , Yamato M, Kanazawa H, Okano T. Poly(N-isopropylacrylamide)-based thermoresponsive surfaces provide new types of biomedical applications. Biomaterials 2018;153:27–48. 29096399 10.1016/j.biomaterials.2017.10.026

[63] Kobayashi J , Kikuchi A, Aoyagi T, Okano T. Cell sheet tissue engineering: cell sheet preparation, harvesting/manipulation, and transplantation. J Biomed Mater Res A 2019;107:955–67. 30684395 10.1002/jbm.a.36627

[64] Teichmann J , Nitschke M, Pette D, Valtink M, Gramm S, Härtel FV, Noll T, Funk RHW, Engelmann K, Werner C. Thermo-responsive cell culture carriers based on poly(vinyl methyl ether)—the effect of biomolecular ligands to balance cell adhesion and stimulated detachment. Sci Technol Adv Mater 2015;16:045003. 27877823 10.1088/1468-6996/16/4/045003 PMC5090182

[65] Sumide T , Nishida K, Yamato M, Ide T, Hayashida Y, Watanabe K, Yang J, Kohno C, Kikuchi A, Maeda N, Watanabe H, Okano T, Tano Y. Functional human corneal endothelial cell sheets harvested from temperature‐responsive culture surfaces. FASEB J 2006;20:392–4. 16339916 10.1096/fj.04-3035fje

[66] Walshe J , Abdulsalam NAK, Suzuki S, Chirila TV, Harkin DG. Growth of human and sheep corneal endothelial cell layers on biomaterial membranes. J Vis Exp 2020;2020:e60762. 10.3791/60762 32090992

[67] Nitschke M , Gramm S, Götze T, Valtink M, Drichel J, Voit B, Engelmann K, Werner C. Thermo-responsive poly(NiPAAm-co-DEGMA) substrates for gentle harvest of human corneal endothelial cell sheets. J Biomed Mater Res A 2007;80:1003–10. 17187393 10.1002/jbm.a.31098

[68] Lai JY , Chen KH, Hsiue GH. Tissue-engineered human corneal endothelial cell sheet transplantation in a rabbit model using functional biomaterials. Transplantation 2007;84:1222–32. 18049106 10.1097/01.tp.0000287336.09848.39

[69] Ide T , Nishida K, Yamato M, Sumide T, Utsumi M, Nozaki T, Kikuchi A, Okano T, Tano Y. Structural characterization of bioengineered human corneal endothelial cell sheets fabricated on temperature-responsive culture dishes. Biomaterials 2006;27:607–14. 16099037 10.1016/j.biomaterials.2005.06.005

[70] Lai JY , Chen KH, Hsu WM, Hsiue GH, Lee YH. Bioengineered human corneal endothelium for transplantation. Arch Ophthalmol 2006;124:1441–8. 17030712 10.1001/archopht.124.10.1441

[71] Götze T , Valtink M, Nitschke M, Gramm S, Hanke T, Engelmann K, Werner C. Cultivation of an immortalized human corneal endothelial cell population and two distinct clonal subpopulations on thermo-responsive carriers, Graefe’s arch. Graefes Arch Clin Exp Ophthalmol 2008;246:1575–83. 18696098 10.1007/s00417-008-0904-6

[72] Shao C , Chen J, Chen P, Zhu M, Yao Q, Gu P, Fu Y, Fan X. Targeted transplantation of human umbilical cord blood endothelial progenitor cells with immunomagnetic nanoparticles to repair corneal endothelium defect. Stem Cells Dev 2015;24:756–67. 25315152 10.1089/scd.2014.0255 PMC4356232

[73] Huhtala A , Pohjonen T, Salminen L, Salminen A, Kaarniranta K, Uusitalo H. In vitro biocompatibility of degradable biopolymers in cell line cultures from various ocular tissues: extraction studies. J Mater Sci Mater Med 2008;19:645–9. 17619963 10.1007/s10856-007-3192-5

[74] Wang TJ , Wang IJ, Chen YH, Lu JN, Young TH. Polyvinylidene fluoride for proliferation and preservation of bovine corneal endothelial cells by enhancing type IV collagen production and deposition. J Biomed Mater Res A 2012;100:252–60. 22042711 10.1002/jbm.a.33274

[75] Mimura T , Yamagami S, Usui T, Ishii Y, Ono K, Yokoo S, Funatsu H, Araie M, Amano S. Long-term outcome of iron-endocytosing cultured corneal endothelial cell transplantation with magnetic attraction. Exp Eye Res 2005;80:149–57. 15670793 10.1016/j.exer.2004.08.021

[76] Rajab TK , O'Malley TJ, Tchantchaleishvili V. Decellularized scaffolds for tissue engineering: current status and future perspective. Artif Organs 2020;44:1031–43. 32279344 10.1111/aor.13701

[77] Bayyoud T , Thaler S, Hofmann J, Maurus C, Spitzer MS, Bartz-Schmidt KU, Szurman P, Yoeruek E. Decellularized bovine corneal posterior lamellae as carrier matrix for cultivated human corneal endothelial cells. Curr Eye Res 2012;37:179–86. 22335804 10.3109/02713683.2011.644382

[78] Zhang C , Du L, Sun P, Shen L, Zhu J, Pang K, Wu X. Construction of tissue-engineered full-thickness cornea substitute using limbal epithelial cell-like and corneal endothelial cell-like cells derived from human embryonic stem cells. Biomaterials 2017;124:180–94. 28199886 10.1016/j.biomaterials.2017.02.003

[79] Yoeruek E , Saygili O, Spitzer MS, Tatar O, Bartz-Schmidt KU, Szurman P. Human anterior lens capsule as carrier matrix for cultivated human corneal endothelial cells. Cornea 2009;28:416–20. 19411960 10.1097/ICO.0b013e31818c2c36

[80] Choi JS , Williams JK, Greven M, Walter KA, Laber PW, Khang G, Soker S. Bioengineering endothelialized neo-corneas using donor-derived corneal endothelial cells and decellularized corneal stroma. Biomaterials 2010;31:6738–45. 20541797 10.1016/j.biomaterials.2010.05.020

[81] Choi JS , Kim EY, Kim MJ, Giegengack M, Khan FA, Khang G, Soker S. In vitro evaluation of the interactions between human corneal endothelial cells and extracellular matrix proteins. Biomed Mater 2013;8:014108. 23353814 10.1088/1748-6041/8/1/014108

[82] Madden PW , Lai JNX, George KA, Giovenco T, Harkin DG, Chirila TV. Human corneal endothelial cell growth on a silk fibroin membrane. Biomaterials 2011;32:4076–84. 21427010 10.1016/j.biomaterials.2010.12.034

[83] Anton-Sales I , D'Antin JC, Fernández-Engroba J, Charoenrook V, Laromaine A, Roig A, Michael R. Bacterial nanocellulose as a corneal bandage material: a comparison with amniotic membrane. Biomater Sci 2020;8:2921–30. 32314754 10.1039/d0bm00083c

[84] Wang TJ , Wang IJ, Chen S, Chen YH, Young TH. The phenotypic response of bovine corneal endothelial cells on chitosan/polycaprolactone blends. Colloids Surf B Biointerfaces 2012;90:236–43. 22078926 10.1016/j.colsurfb.2011.10.043

[85] Wang TJ , Wang IJ, Lu JN, Young TH. Novel chitosan-polycaprolactone blends as potential scaffold and carrier for corneal endothelial transplantation. Mol Vis 2012;18:255–64. 22328821 PMC3276373

[86] Young TH , Wang IJ, Hu FR, Wang TJ. Fabrication of a bioengineered corneal endothelial cell sheet using chitosan/polycaprolactone blend membranes. Colloids Surf B Biointerfaces 2014;116:403–10. 24531150 10.1016/j.colsurfb.2014.01.024

[87] Wang YH , Young TH, Wang TJ. Investigating the effect of chitosan/polycaprolactone blends in differentiation of corneal endothelial cells and extracellular matrix compositions. Exp Eye Res 2019;185:107679. 31129253 10.1016/j.exer.2019.05.019

[88] Ozcelik B , Brown KD, Blencowe A, Daniell M, Stevens GW, Qiao GG. Ultrathin chitosan–poly(ethylene glycol) hydrogel films for corneal tissue engineering. Acta Biomater 2013;9:6594–605. 23376126 10.1016/j.actbio.2013.01.020

[89] Ozcelik B , Brown KD, Blencowe A, Ladewig K, Stevens GW, Scheerlinck JPY, Abberton K, Daniell M, Qiao GG. Biodegradable and biocompatible poly(ethylene glycol)-based hydrogel films for the regeneration of corneal endothelium. Adv Healthc Mater 2014;3:1496–507. 24652807 10.1002/adhm.201400045

[90] Van Hoorick J , Delaey J, Vercammen H, Van Erps J, Thienpont H, Dubruel P, Zakaria N, Koppen C, Van Vlierberghe S, Van den Bogerd B. Designer Descemet membranes containing PDLLA and functionalized gelatins as corneal endothelial scaffold. Adv Healthcare Mater 2020;9:2000760. 10.1002/adhm.202000760 32603022

[91] Lai JY , Cheng HY, Hui-Kang Ma D. Investigation of overrun-processed porous hyaluronic acid carriers in corneal endothelial tissue engineering. PLoS One 2015;10:e0136067. 26296087 10.1371/journal.pone.0136067 PMC4546624

[92] Lai JY. Influence of pre-freezing temperature on the corneal endothelial cytocompatibility and cell delivery performance of porous hyaluronic acid hydrogel carriers. Int J Mol Sci 2015;16:18796–811. 26270663 10.3390/ijms160818796 PMC4581272

[93] Lu PL , Lai JY, Ma DHK, Hsiue GH. Carbodiimide cross-linked hyaluronic acid hydrogels as cell sheet delivery vehicles: characterization and interaction with corneal endothelial cells. J Biomater Sci Polym Ed 2008;19:1–18. 18177550 10.1163/156856208783227695

[94] Liang Y , Liu W, Han B, Yang C, Ma Q, Zhao W, Rong M, Li H. Fabrication and characters of a corneal endothelial cells scaffold based on chitosan. J Mater Sci Mater Med 2011;22:175–83. 21107657 10.1007/s10856-010-4190-6

[95] Chen J , Li Q, Xu J, Huang Y, Ding Y, Deng H, Zhao S, Chen R. Study on biocompatibility of complexes of collagen-chitosan-sodium hyaluronate and cornea. Artif Organs 2005;29:104–13. 15670279 10.1111/j.1525-1594.2005.29021.x

[96] Gao X , Liu W, Han B, Wei X, Yang C. Preparation and properties of a chitosan-based carrier of corneal endothelial cells. J Mater Sci Mater Med 2008;19:3611–9. 18642061 10.1007/s10856-008-3508-0

[97] Xu W , Wang Z, Li T, Wang L, Zhang W, Liang Y, Liu C. Membranes based on carboxymethyl chitin as potential scaffolds for corneal endothelial transplantation. Polym J 2017;49:789–98.

[98] Lai JY. Biocompatibility of genipin and glutaraldehyde cross-linked chitosan materials in the anterior chamber of the eye. Int J Mol Sci 2012;13:10970–85. 23109832 10.3390/ijms130910970 PMC3472724

[99] Yoshida J , Oshikata-Miyazaki A, Yokoo S, Yamagami S, Takezawa T, Amano S. Development and evaluation of porcine atelocollagen vitrigel membrane with a spherical curve and transplantable artificial corneal endothelial grafts. Invest Ophthalmol Vis Sci 2014;55:4975–81. 25028359 10.1167/iovs.14-14211

[100] Yoshida J , Yokoo S, Oshikata-Miyazaki A, Amano S, Takezawa T, Yamagami S. Transplantation of human corneal endothelial cells cultured on Bio-Engineered collagen vitrigel in a rabbit model of corneal endothelial dysfunction. Curr Eye Res 2017;42:1420–5. 28933958 10.1080/02713683.2017.1351568

[101] Levis HJ , Peh GSL, Toh KP, Poh R, Shortt AJ, Drake RAL, Mehta JS, Daniels JT. Plastic compressed collagen as a novel carrier for expanded human corneal endothelial cells for transplantation. PLoS One 2012;7:e50993. 23226443 10.1371/journal.pone.0050993 PMC3511456

[102] Cen YJ , Feng Y. Constructing a novel three-dimensional biomimetic corneal endothelium graft by culturing corneal endothelium cells on compressed collagen gels. Chin Med J (Engl) 2018;131:1710–4. 29998891 10.4103/0366-6999.235883 PMC6048920

[103] Lai JY , Lu PL, Chen KH, Tabata Y, Hsiue GH. Effect of charge and molecular weight on the functionality of gelatin carriers for corneal endothelial cell therapy. Biomacromolecules 2006;7:1836–44. 16768405 10.1021/bm0601575

[104] Niu G , Choi JS, Wang Z, Skardal A, Giegengack M, Soker S. Heparin-modified gelatin scaffolds for human corneal endothelial cell transplantation. Biomaterials 2014;35:4005–14. 24508079 10.1016/j.biomaterials.2014.01.033

[105] Watanabe R , Hayashi R, Kimura Y, Tanaka Y, Kageyama T, Hara S, Tabata Y, Nishida K. A novel gelatin hydrogel carrier sheet for corneal endothelial transplantation. Tissue Eng Part A 2011;17:2213–9. 21534849 10.1089/ten.TEA.2010.0568

[106] Kong B , Sun W, Chen G, Tang S, Li M, Shao Z, Mi S. Tissue-engineered cornea constructed with compressed collagen and laser-perforated electrospun mat. Sci. Rep 2017;7. doi:10.1038/s41598-017-01072-0. PMC5430529 28428541

[107] Spinozzi D , Miron A, Bruinsma M, Dapena I, Lavy I, Binder PS, Rafat M, Oellerich S, Melles GRJ. Evaluation of the suitability of biocompatible carriers as artificial transplants using cultured porcine corneal endothelial cells. Curr Eye Res 2019;44:243–9. 30339045 10.1080/02713683.2018.1536215

[108] Natu MV , Gaspar MN, Fontes Ribeiro CA, Cabrita AM, De Sousa HC, Gil MH. In vitro and in vivo evaluation of an intraocular implant for glaucoma treatment. Int J Pharm 2011;415:73–82. 21641984 10.1016/j.ijpharm.2011.05.047

[109] Natu MV , Gaspar MN, Ribeiro CAF, Correia IJ, Silva D, De Sousa HC, Gil MH. A poly(ε-caprolactone) device for sustained release of an anti-glaucoma drug. Biomed Mater 2011;6:025003. 21293056 10.1088/1748-6041/6/2/025003

[110] Natu MV , Sardinha JP, Correia IJ, Gil MH. Controlled release gelatin hydrogels and lyophilisates with potential application as ocular inserts. Biomed Mater 2007;2:241–9. 18458481 10.1088/1748-6041/2/4/006

[111] Koda S , Okumura N, Kitano J, Koizumi N, Tabata Y. Development of poly lactic/glycolic acid (PLGA) microspheres for controlled release of rho-associated kinase inhibitor. J Ophthalmol 2017;2017. doi:10.1155/2017/1598218. PMC5551544 28819566

[112] Kinoshita S , Koizumi N, Ueno M, Okumura N, Imai K, Tanaka H, Yamamoto Y, Nakamura T, Inatomi T, Bush J, Toda M, Hagiya M, Yokota I, Teramukai S, Sotozono C, Hamuro J. Injection of cultured cells with a ROCK inhibitor for bullous keratopathy. N Engl J Med 2018;378:995–1003. 29539291 10.1056/NEJMoa1712770

[113] Lee MC , Kim DK, Lee OJ, Kim JH, Ju HW, Lee JM, Moon BM, Park HJ, Kim DW, Kim SH, Park CH. Fabrication of silk fibroin film using centrifugal casting technique for corneal tissue engineering. J Biomed Mater Res B Appl Biomater 2016;104:508–14. 25939800 10.1002/jbm.b.33402

[114] Silva MF , de Moraes MA, Nogueira GM, Rodas ACD, Higa OZ, Beppu MM. Glycerin and ethanol as additives on silk fibroin films: insoluble and malleable films. J Appl Polym Sci 2013;128:115–22.

[115] Song JE , Sim BR, Jeon YS, Kim HS, Shin EY, Carlomagno C, Khang G. Characterization of surface modified glycerol/silk fibroin film for application to corneal endothelial cell regeneration. J Biomater Sci Polym Ed 2019;30:263–75. 30324858 10.1080/09205063.2018.1535819

[116] Seow WY , Kandasamy K, Peh GSL, Mehta JS, Sun W. Ultrathin, strong, and cell-adhesive agarose-based membranes engineered as substrates for corneal endothelial cells. ACS Biomater Sci Eng 2019;5:4067–76. 33448808 10.1021/acsbiomaterials.9b00610

[117] Kennedy S , Lace R, Carserides C, Gallagher AG, Wellings DA, Williams RL, Levis HJ. Poly-ε-lysine based hydrogels as synthetic substrates for the expansion of corneal endothelial cells for transplantation. J Mater Sci Mater Med 2019;30:1–13. 10.1007/s10856-019-6303-1 PMC6726667 31485761

[118] Kim DK , Sim BR, Kim JI, Khang G. Functionalized silk fibroin film scaffold using β-Carotene for cornea endothelial cell regeneration. Colloids Surf B Biointerfaces 2018;164:340–6. 29413615 10.1016/j.colsurfb.2017.11.052

[119] Choi JH , Jeon H, Song JE, Oliveira JM, Reis RL, Khang G. Biofunctionalized lysophosphatidic acid/silk fibroin film for cornea endothelial cell regeneration. Nanomaterials 2018;8. doi:10.3390/nano8050290. PMC5977304 29710848

[120] Kimoto M , Shima N, Yamaguchi M, Hiraoka Y, Amano S, Yamagami S. Development of a bioengineered corneal endothelial cell sheet to fit the corneal curvature. Invest Ophthalmol Vis Sci 2014;55:2337–43. 24651553 10.1167/iovs.13-13167

[121] Rizwan M , Peh GSL, Ang HP, Lwin NC, Adnan K, Mehta JS, Tan WS, Yim EKF. Sequentially-crosslinked bioactive hydrogels as nano-patterned substrates with customizable stiffness and degradation for corneal tissue engineering applications. Biomaterials 2017;120:139–54. 28061402 10.1016/j.biomaterials.2016.12.026

[122] Salehi S , Fathi M, Javanmard SH, Bahners T, Gutmann JS, Ergün S, Steuhl KP, Fuchsluger TA. Generation of PGS/PCL blend nanofibrous scaffolds mimicking corneal stroma structure. Macromol Mater Eng 2014;299:455–69.

[123] Salehi S , Czugala M, Stafiej P, Fathi M, Bahners T, Gutmann JS, Singer BB, Fuchsluger TA. Poly (glycerol sebacate)-poly (ε-caprolactone) blend nanofibrous scaffold as intrinsic bio- and immunocompatible system for corneal repair. Acta Biomater 2017;50:370–80. 28069498 10.1016/j.actbio.2017.01.013

[124] Chen J , Yan C, Zhu M, Yao Q, Shao C, Lu W, Wang J, Mo X, Gu P, Fu Y, Fan X. Electrospun nanofibrous SF/P(LLA-CL) membrane: a potential substratum for endothelial keratoplasty. Int J Nanomedicine 2015;10:3337–50. 26005345 10.2147/IJN.S77706 PMC4427599

[125] Muhammad R , Peh GSL, Adnan K, Law JBK, Mehta JS, Yim EKF. Micro- and nano-topography to enhance proliferation and sustain functional markers of donor-derived primary human corneal endothelial cells. Acta Biomater 2015;19:138–48. 25796353 10.1016/j.actbio.2015.03.016

[126] Teo BKK , Goh KJ, Ng ZJ, Koo S, Yim EKF. Functional reconstruction of corneal endothelium using nanotopography for tissue-engineering applications. Acta Biomater 2012;8:2941–52. 22522131 10.1016/j.actbio.2012.04.020

[127] Koo S , Muhammad R, Peh GSL, Mehta JS, Yim EKF. Micro- and nanotopography with extracellular matrix coating modulate human corneal endothelial cell behavior. Acta Biomater 2014;10:1975–84. 24456758 10.1016/j.actbio.2014.01.015

[128] Zhou C , Lei F, Chodosh J, Paschalis EI. The role of titanium surface microtopography on adhesion, proliferation, transformation, and matrix deposition of corneal cells. Invest Ophthalmol Vis Sci 2016;57:1927–38. 27092719 10.1167/iovs.15-18406

[129] Seow WY , Kandasamy K, Purnamawati K, Sun W, Hauser CAE. Thin peptide hydrogel membranes suitable as scaffolds for engineering layered biostructures. Acta Biomater 2019;88:293–300. 30721784 10.1016/j.actbio.2019.02.001

[130] Bhatia SK. Tissue engineering for clinical applications. Biotechnol J 2010;5:1309–23. 21154670 10.1002/biot.201000230

[131] Nourian Dehkordi A , Mirahmadi Babaheydari F, Chehelgerdi M, Raeisi Dehkordi S. Skin tissue engineering: wound healing based on stem-cell-based therapeutic strategies. Stem Cell Res. Ther 2019;10:1–20. 30922387 10.1186/s13287-019-1212-2 PMC6440165

[132] Sierra-Sánchez Á , Kim KH, Blasco-Morente G, Arias-Santiago S. Cellular human tissue-engineered skin substitutes investigated for deep and difficult to heal injuries. NPJ Regen Med 2021;61:1–23. 10.1038/s41536-021-00144-0 PMC8211795 34140525

[133] Vinatier C , Guicheux J. Cartilage tissue engineering: from biomaterials and stem cells to osteoarthritis treatments. Ann Phys Rehabil Med 2016;59:139–44. 27079583 10.1016/j.rehab.2016.03.002

[134] Jiang S , Guo W, Tian G, Luo X, Peng L, Liu S, Sui X, Guo Q, Li X. Clinical application status of articular cartilage regeneration techniques: tissue-engineered cartilage brings new hope. Stem Cells Int 2020;2020:5690252. 32676118 10.1155/2020/5690252 PMC7345961

[135] Zhang PX , Han N, Kou YH, Zhu QT, Liu XL, Quan DP, Chen JG, Jiang BG. Tissue engineering for the repair of peripheral nerve injury. Neural Regen Res 2019;14:51–8. 30531070 10.4103/1673-5374.243701 PMC6263012

[136] Rayner MLD , Day AGE, Bhangra KS, Sinden J, Phillips JB. Engineered neural tissue made using clinical-grade human neural stem cells supports regeneration in a long gap peripheral nerve injury model. Acta Biomater 2021;135:203–13. 34455110 10.1016/j.actbio.2021.08.030

[137] Boni R , Ali A, Shavandi A, Clarkson AN. Current and novel polymeric biomaterials for neural tissue engineering. J Biomed Sci 2018;25:1–21. 30572957 10.1186/s12929-018-0491-8 PMC6300901

[138] Sorkio A , Koch L, Koivusalo L, Deiwick A, Miettinen S, Chichkov B, Skottman H. Human stem cell based corneal tissue mimicking structures using laser-assisted 3D bioprinting and functional bioinks. Biomaterials 2018;171:57–71. 29684677 10.1016/j.biomaterials.2018.04.034

[139] Jiang X , Peng Y, Yang C, Liu W, Han B. The feasibility study of an in situ marine polysaccharide-based hydrogel as the vitreous substitute. J Biomed Mater Res A 2018;106:1997–2006. 29569838 10.1002/jbm.a.36403

[140] Liang Y , Liu W, Han B, Yang C, Ma Q, Song F, Bi Q. An in situ formed biodegradable hydrogel for reconstruction of the corneal endothelium. Colloids Surf B Biointerfaces 2011;82:1–7. 20832263 10.1016/j.colsurfb.2010.07.043

